# Preventing dataset shift from breaking machine-learning biomarkers

**DOI:** 10.1093/gigascience/giab055

**Published:** 2021-09-28

**Authors:** Jérôme Dockès, Gaël Varoquaux, Jean-Baptiste Poline

**Affiliations:** McGill University, 845 Sherbrooke St W, Montreal, Quebec H3A 0G4, Canada; McGill University, 845 Sherbrooke St W, Montreal, Quebec H3A 0G4, Canada; INRIA; McGill University, 845 Sherbrooke St W, Montreal, Quebec H3A 0G4, Canada

**Keywords:** biomarker, machine learning, generalization, dataset shift

## Abstract

Machine learning brings the hope of finding new biomarkers extracted from cohorts with rich biomedical measurements. A good biomarker is one that gives reliable detection of the corresponding condition. However, biomarkers are often extracted from a cohort that differs from the target population. Such a mismatch, known as a dataset shift, can undermine the application of the biomarker to new individuals. Dataset shifts are frequent in biomedical research, e.g.,  because of recruitment biases. When a dataset shift occurs, standard machine-learning techniques do not suffice to extract and validate biomarkers. This article provides an overview of when and how dataset shifts break machine-learning–extracted biomarkers, as well as detection and correction strategies.

## Introduction: Dataset Shift Breaks Learned Biomarkers

Biomarkers are measurements that provide information about a medical condition or physiological state [1]. For example, the presence of an antibody may indicate an infection; a complex combination of features extracted from a medical image can help assess the evolution of a tumor. Biomarkers are important for diagnosis, prognosis, and treatment or risk assessments.

Complex biomedical measures may carry precious medical information, as with histopathological images or genome sequencing of biopsy samples in oncology. Identifying quantitative biomarkers from these requires sophisticated statistical analysis. With large datasets becoming accessible, supervised machine learning provides new promise by optimizing the information extracted to relate to a specific output variable of interest, such as a cancer diagnosis [2–4]. These methods, cornerstones of artificial intelligence, are starting to appear in clinical practice: a machine-learning–based radiological tool for breast cancer diagnosis has recently been approved by the U.S. Food and Drug Administration [5].

Can such predictive biomarkers, extracted through complex data processing, be safely used in clinical practice, beyond the initial research settings? One risk is the potential mismatch, or “dataset shift," between the distribution of the individuals used to estimate this statistical link and that of the target population that should benefit from the biomarker. In this case, the extracted associations may not apply to the target population [6]. Computer-aided diagnostics of thoracic diseases from X-ray images has indeed been shown to be unreliable for individuals of a given sex if built from a cohort over-representing the other sex [7]. More generally, machine-learning systems may fail on data from different imaging devices, hospitals, populations with a different age distribution, and so forth. Dataset biases are in fact frequent in medicine. For instance selection biases—e.g., due to volunteering self-selection, non-response, or dropout [8, 9]—may cause cohorts to capture only a small range of possible patients and disease manifestations in the presence of spectrum effects [10, 11]. Dataset shift or dataset bias can cause systematic errors that cannot be fixed by acquiring larger datasets and require specific methodological care.

In this article, we consider predictive biomarkers identified with supervised machine learning. We characterize the problem of dataset shift, show how it can hinder the use of machine learning for health applications [12, 13], and provide mitigation strategies.

## A Primer on Machine Learning for Biomarkers

### Empirical risk minimization

Let us first introduce the principles of machine learning used to identify biomarkers. Supervised learning captures, from observed data, the link between a set of input measures (features) *X* and an output (e.g.,  a condition) *Y*: e.g., the relation between the absorption spectrum of oral mucosa and blood glucose concentration [14]. A supervised learning algorithm finds a function *f* such that *f*(*X*) is as close as possible to the output *Y*. Following machine-learning terminology, we call the system’s best guess *f*(*X*) for a value *X* a “prediction," even when it does not concern a measurement in the future.

Empirical risk minimization, central to machine learning, uses a loss function *L* to measure how far a prediction *f*(*X*) is from the true value *Y*, e.g., the squared difference:

(1) \begin{eqnarray*}
L(Y, f(X)) = (Y - f(X))^2 . \end{eqnarray*}

The goal is to find a function *f* that has a small “risk," which is the expected loss on the true distribution of *X* and *Y*, i.e.,  on unseen individuals. The true risk cannot be computed in practice: it would require having seen all possible patients, the true distribution of patients. The empirical risk is used instead: the average error over available examples,

(2) \begin{eqnarray*}
\hat{R}(f) = \frac{1}{n}\sum \nolimits_{i=1}^n L(y_i, f(x_i)), \end{eqnarray*}

where $\lbrace (x_i, y_i)\, ,\, i=1,\dots ,n\rbrace$ are available (*X, Y*) data, called “training" examples. The statistical link of interest is then approximated by choosing *f* within a family of candidate functions as the one that minimizes the empirical risk $\hat{R}(f)$.

The crucial assumption underlying this very popular approach is that the prediction function *f* will then be applied to individuals drawn from the same population as the training examples {*x_i_*, *y_i_*}. It can be important to distinguish the source data, used to fit and evaluate a machine-learning model (e.g., a dataset collected for research), from the target data, on which predictions are meant to be used for clinical applications (e.g., new visitors to a hospital). Indeed, if the training examples are not representative of the target population—if there is a dataset shift—the empirical risk is a poor estimate of the expected error, and *f* will not perform well on individuals from the target population.

### Evaluation: Independent test set and cross-validation

Once a model has been estimated from training examples, measuring its error on these same individuals results in a (sometimes wildly) optimistic estimate of the expected error on *unseen* individuals (Friedman et al. [15], Sec. 7.4, Poldrack et al. [16], Sec. 1, “Association vs Prediction”). Indeed, predictors chosen from a rich family of functions are very flexible and can learn rules that fit the training examples tightly but fail to generalize to new individuals. This is called “overfitting."

To obtain valid estimates of the expected performance on new data, the error is measured on an independent sample held out during training, called the test set. The most common approach to obtain such a test set is to randomly split the available data. This process is usually repeated with several splits, a procedure called cross-validation ([15, Sec. 7, 17]).

When training and test examples are chosen uniformly from the same sample, they are drawn from the same distribution (i.e., the same population): there is no dataset shift. Some studies also measure the error on an independent dataset (e.g., [18, 19]). This helps establish external validity, assessing whether the predictor will perform well outside of the dataset used to define it [20]. Unfortunately, the biases in participant recruitment may be similar in independently collected datasets. For example if patients with severe symptoms are difficult to recruit, this is likely to distort all datasets similarly. Testing on a dataset collected independently is therefore a useful check but no silver bullet to rule out dataset shift issues.

## False Solutions to Tackling Dataset Shift

We now discuss some misconceptions and confusions with problems not directly related to dataset shift.

### “Deconfounding” does not correct dataset shift for predictive models

Dataset shift is sometimes confused with the notion of confounding because both settings arise from an undesired effect in the data. Confounding comes from causal analysis, estimating the effect of a treatment—an intervention, sometimes fictional—on an outcome. A confounder is a third variable—e.g., age or a comorbidity—that influences both the treatment and the outcome. It can produce a non-causal association between the two (see [21], Chap. 7, for a precise definition). However, the machine-learning methods that we consider here capture statistical associations but do not target causal effects. Indeed, for biomarkers, the association itself is interesting, whether causal or not. Elevated body temperature may be the consequence of a condition but also cause a disorder. It is a clinically useful measure in both settings.

**Figure 1:**
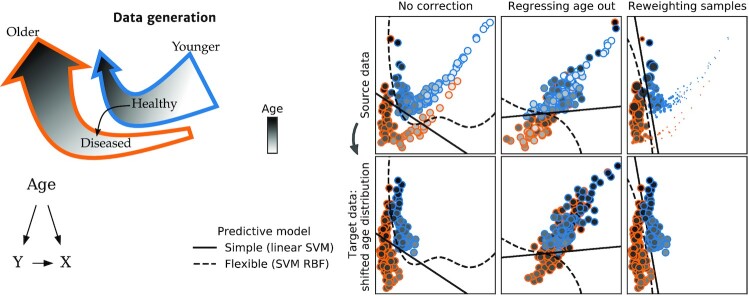
Classification with dataset shift—regressing out a correlate of the shift does not help generalization. The task is to classify patients (orange) from healthy controls (blue), using 2D features. Age, indicated by the shade of gray, influences both the features and the probability of disease. *Left:* Generative process for the simulated data. Age influences both the target *Y* and the features *X*, and *Y* also has an effect on *X*. Between the source and target datasets, the distribution of age changes. The 2 arrows point towards increasing age and represent the Healthy and Diseased populations, corresponding to the orange and blue clouds of points in the right panel. The grayscale gradient in the arrows represents the increasing age of the individuals (older individuals correspond to a darker shade). Throughout their life, individuals can jump from the Healthy trajectory to the Diseased trajectory, which is slightly offset in this 2D feature space. As age increases, the prevalence of the disease increases, hence the Healthy trajectory contains more individuals of young ages (its wide end) and fewer at older ages (its narrow end)—and vice versa for the Diseased trajectory. *Right:* Predictive models. In the target data (bottom row), the age distribution is shifted: individuals tend to be older. Elderly individuals are indeed often less likely to participate in clinical studies [24]. *First column:* No correction is applied. As the situation is close to a covariate shift (see Section “Covariate shift"), a powerful learner (RBF-SVM) generalizes well to the target data. An over-constrained model—Linear-SVM—generalizes poorly. *Second column:* Wrong approach. To remove associations with age, features are replaced by the residuals after regressing them on age. This destroys the signal and results in poor performance for both models and datasets. *Third column:* Samples are weighted to give more importance to those more likely in the target distribution. Small circles indicate younger individuals, with less influence on the classifier estimation. This reweighting improves prediction for the linear model on the older population. AUC: area under the curve.

**Figure 2:**
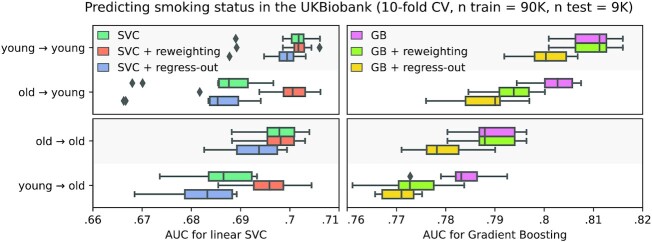
Predicting the smoking status of UKBiobank participants. Different predictive models are trained on 90,000 UKBiobank participants and tested on 9,000 participants with a possibly shifted age distribution. “Young → old” means the training set was drawn from a younger sample than the testing set. Models perform better when trained on a sample drawn from the same population as the testing set. Reweighting examples that are more likely in the test distribution (“+ reweighting” strategy, known as Importance Weighting, see Section “Importance Weighting”) alleviates the issue for the simple linear model but is detrimental for the gradient boosting. Regressing out the age (“+ regress-out” strategy) is a bad idea and degrades prediction performance in all configurations. The boxes represent the first, second and third quartiles of scores across cross-validation folds. Whiskers represent the rest of the distribution, except for outliers, defined as points beyond 1.5 times the IQR past the low and high quartiles, and represented with diamond fliers.

Tools for causal analysis are not all useful for prediction, as pointed out by seminal textbooks: “if the goal of the data analysis is purely predictive, no adjustment for confounding is necessary [...] the concept of confounding does not even apply.” ([21], Sec. 18.1), or Pearl [22]. In prediction settings, applying procedures meant to adjust for confounding generally degrades prediction performance without solving the dataset shift issue. Figure 1 demonstrates the detrimental effect of “deconfounding” on simulated data: while the target population is shifted due to a different age distribution, removing the effect of age also removes the separation between the 2 outcomes of interest. The same behavior is visible on real epidemiologic data with age shifts, such as predicting the smoking status of participants in the UKBiobank study [23], as shown in Fig. 2. Drawing training and testing samples with different age distributions highlights the effect of these age shifts on prediction performance (see Appendix B, “Tobacco smoking prediction in the UKBiobank" for details on the procedure). For a given learner and test population, training on a different population degrades prediction. For example, predictions on the old population are degraded when the model is trained on the young population. A flexible model (gradient boosting) outperforms the linear model with or without dataset shift. “Regressing out” the age (as in the second column of Fig. 1, “+ regress-out” strategy in Fig. 2) degrades the predictions in *all* configurations.

For both illustrations on simulated and real data (Fig. 1 and 2), we also demonstrate an approach suitable for predictive models: reweighting training examples giving more importance to those more likely in the test population. This approach improves the predictions of the overconstrained (misspecified) linear model in the presence of dataset shift but degrades the predictions of the powerful learner. The non-linear model already captures the correct separation for both young and old individuals; thus reweighting examples does not bring any benefit but only increases the variance of the empirical risk. A more detailed discussion of this approach, called “importance weighting," is provided in Section “Importance weighting: a generic tool against dataset shift" .

### Training examples should not be selected to be homogeneous

To obtain valid predictive models that perform well beyond the training sample, it is crucial to collect datasets that represent the whole population and reflect its diversity as much as possible [6, 25, 26]. Yet clinical research often emphasizes the opposite: very homogeneous datasets and carefully selected participants. While this may help reduce variance and improve statistical testing, it degrades prediction performance and fairness. In other words, the machine-learning system may perform worse for segments of the population that are under-represented in the dataset, resulting in uneven quality of care if it is deployed in clinical settings. Therefore in predictive settings, where the goal is machine-learning models that generalize well, large and diverse datasets are desirable.

### Simpler models are not less sensitive to dataset shift

Often, flexible models can be more robust to dataset shifts, and thus generalize better, than linear models [27], as seen in Figs 1 and 2. Indeed, an over-constrained (ill-specified) model may only fit well a restricted region of the feature space, and its performance can degrade if the distribution of inputs changes, even if the relation to the output stays the same (i.e.,  when covariate shift occurs, see Section “Covariate shift" ).

Dataset shift does not call for simpler models because it is not a small-sample issue. Collecting more data from the same sources will not correct systematic dataset bias.

## Preferential Sample Selection: A Common Source of Shift

In 2017, competitors in the million-dollar-prize Data Science Bowl [28] used machine learning to predict whether individuals would receive a diagnosis of lung cancer within 1 year, on the basis of a computed tomographic (CT) scan. Assuming that the winning model achieves satisfying accuracy on left-out examples from this dataset, is it ready to be deployed in hospitals? Most likely not. Selection criteria may make this dataset not representative of the potential lung cancer patients general population. Selected participants verified many criteria, including being a smoker and not having recent medical problems such as pneumonia. How would the winning predictor perform on a more diverse population? For example, another disease could present features that the classifier could mistakenly take for signs of lung cancer. Beyond explicit selection criteria, many factors such as age, ethnicity, or socioeconomic status influence participation in biomedical studies [24, 29–31]. Not only can these shifts reduce overall predictive performance, they can also lead to discriminative clinical decisions for poorly represented populations [32–35,80].

The examples above are instances of preferential selection, which happens when members of the population of interest do not have equal probabilities of being included in the source dataset: the selection *S* is not independent of (*X, Y*). Preferential sample selection is ubiquitous and cannot always be prevented by careful study design [36]. It is therefore a major challenge to the identification of reliable and fair biomarkers. Beyond preferential sample selection, there are many other sources of dataset shifts, e.g., population changes over time, interventions such as the introduction of new diagnostic codes in Electronic Health Records [37], and the use of different acquisition devices.

### The selection mechanism influences the type of dataset shift

The correction for a dataset shift depends on the nature of this shift, characterized by which and how distributions are modified [27]. Knowledge of the mechanism producing the dataset shift helps formulate hypotheses about distributions that remain unchanged in the target data ([38, 39], Chap. 5).

**Figure 3:**
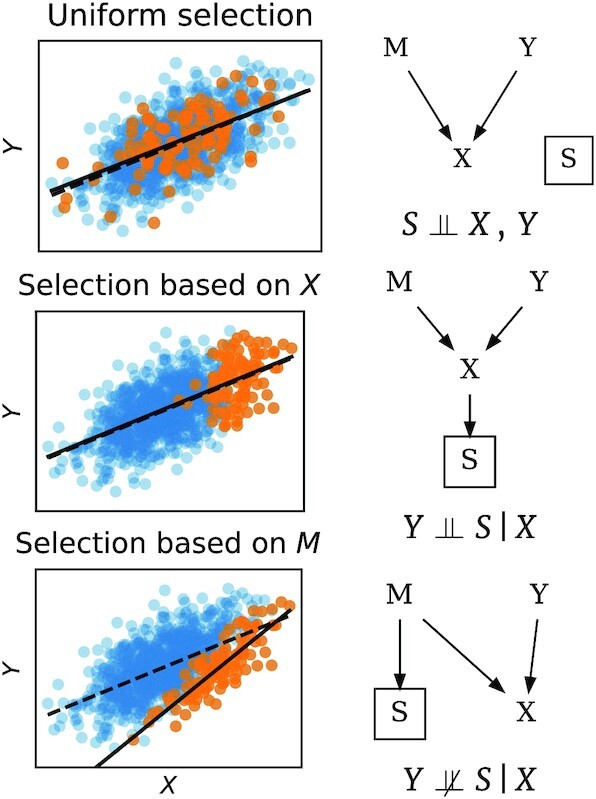
Sample selection bias: three examples. On the right are graphs giving conditional independence relations [40]. *Y* is the lesion volume to be predicted (i.e., the output). *M* are the imaging parameters, e.g., contrast agent dosage. *X* is the image, and depends both on *Y* and *M* (in this toy example *X* is computed as $X := Y + M + \epsilon$, where ϵ is additive noise). *S* indicates that data are selected to enter the source dataset (orange points) or not (blue points). The symbol ${\perp \!\!\! \perp }$ means independence between variables. Preferentially selecting samples results in a dataset shift (middle and bottom row). Depending on whether $Y {\perp \!\!\! \perp }\ S \, |\, X$, the conditional distribution of $Y \, |\, X$—here lesion volume given the image—estimated on the selected data may be biased or not.

Figure 3 illustrates this process with a simulated example of preferential sample selection. We consider the problem of predicting the volume *Y* of a tumor from features *X* extracted from contrast CT images. These features can be influenced not only by the tumor size but also by the dosage of a contrast agent *M*. The top panel of Fig. 3 shows a selection of data independent of the image and tumor volume: there is no dataset shift. In the second panel, selection depends on the CT image itself (e.g., images with a low signal-to-noise ratio are discarded). As selection is independent of the tumor volume *Y* given the image *X*, the distribution of images changes but the conditional distribution $P(Y \, |\, X)$ stays the same: we face a “covariate shift" (see Section “Covariate shift"). The learned association remains valid. Moreover, reweighting examples to give more importance to those less likely to be selected can improve predictions for target data (Section “Importance Weighting"), and it can be done with only unlabeled examples from the target data. In the bottom panel, individuals who received a low dose of contrast agent are less likely to enter the training dataset. Selection is therefore not independent of tumor volume (the output) given the image values (the input features). Therefore we have sample selection bias: the relation $P(Y \, |\, X)$ is different in source and target data, which will affect the performance of the prediction.

As these examples illustrate, the causal structure of the data helps identify the type of dataset shift and what information is needed to correct it. When such information is available, it may be possible to leverage it to improve robustness to dataset shift (e.g.,  [41]).

## Importance Weighting: A Generic Tool Against Dataset Shift

Importance weighting is a simple approach to dataset shift that applies to many situations and can be easy to implement.

Dataset shift occurs when the joint distribution of the features and outputs is different in the source (data used to fit the machine-learning model) and in the target data. Informally, importance weighting consists in reweighting the available data to create a pseudo-sample that follows the same distribution as the target population.

To do so, examples are reweighted by their “importance weights"—the ratio of their likelihood in target data over source data. Examples that are rare in the source data but are likely in the target data are more relevant and therefore receive higher weights. A related approach is “importance sampling"—resampling the training data according to the importance weights. Many statistical learning algorithms—including Support Vector Machines (SVM), decision trees, random forests, and neural networks—naturally support weighting the training examples. Therefore, the challenge lies mostly in the estimation of the appropriate sample weights and the learning algorithm itself does not need to be modified.

**Figure 4:**
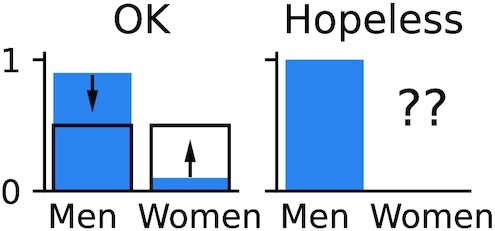
Dataset shifts that may or may not be compensated by reweighting. *Left:* Distribution of sex can be balanced by downweighting men and upweighting women. *Right:* Women are completely missing; the dataset shift cannot be fixed by importance weighting.

To successfully use importance weighting, no part of the target distribution should be completely unseen. For example, if sex (among other features) is used to predict heart failure and the dataset only includes men, importance weighting cannot transform this dataset and make its sex distribution similar to that of the general population (Fig. 4). Conversely, the source distribution may be broader than the target distribution (as seen, e.g., in Fig. 1).

Importance weights can also be applied to validation examples, which may produce a more accurate estimation of generalization error on target data.

Importance weighting is a well-known approach, and an important body of literature focuses on its application and the estimation of importance weights. It is illustrated on small datasets for the prediction of breast cancer in Dudík et al. [42] and heart disease in Kouw and Loog [43]. However, it cannot always be applied: some knowledge of the target distribution is required, and the source distribution must cover its support. Moreover, importance weighting can increase the variance of the empirical risk estimate, and thus sometimes degrades performance, as seen in Fig. 2. It is therefore a straightforward and popular approach to consider but not a complete solution. It is particularly beneficial when using a simple learning model that cannot capture the full complexity of the data, such as the linear models in Fig. 1. Indeed, simple models are often preferred in biomedical applications because they are easy to interpret and audit.

In Appendix A, “Definition and Estimation of Importance Weights", we provide a more precise definition of the importance weights, as well as an overview of how they can be estimated and used.

## Other Approaches to Dataset Shift

Beyond importance weighting, many other solutions to dataset shift have been proposed. They are typically more difficult to implement because they require adapting or desiging new learning algorithms. However, they may be more effective, or applicable when information about the target distribution is lacking. We summarize a few of these approaches here. A more systematic review can be found in Kouw and Loog [43]. Weiss et al. [44] and Pan and Yang [45] give systematic reviews of transfer learning (a wider family of learning problems that includes dataset shift).

The most obvious solution is to do nothing, ignoring the dataset shift. This approach should be included as a baseline when testing on a sample of target data, which is a prerequisite to clinical use of a biomarker [12, 27]. With flexible models, this is a strong baseline that can outperform importance weighting, as in the right panel of Fig. 2.

Another approach is to learn representations—transformations of the signal—that are invariant to the shift [46]. Some deep-learning methods strive to extract features that are predictive of the target while having similar distributions in the source and target domains (e.g., [47]), or while preventing an adversary from distinguishing source and target data (“domain-adversarial” learning, e.g., [48]). When considering such methods, one must be aware of the fallacy shown in Fig. 1: making the features invariant to the effect driving the dataset shift can remove valuable signal if this effect is not independent of the outcome of interest.

It may also be possible to explicitly model the mapping from source to target domains, e.g., by training a neural network to translate images from one modality or imaging device to another, or by relying on optimal transport [49].

Finally, synthetic data augmentation sometimes helps—relying on known invariances, e.g., for images by applying affine transformations, resampling, etc. , or with learned generative models (e.g., [50]).

### Performance heterogeneity and fairness

It can be useful not to target a specific population but rather find a predictor robust to certain dataset shifts. Distributionally robust optimization tackles this goal by defining an ambiguity, or uncertainty set—a set of distributions to which the target distribution might belong—then minimizing the worse risk across all distributions in this set (see [51] for a review). The uncertainty set is often chosen centered on the empirical (source) distribution for some divergence between distributions. Popular choices for this divergence are the Wasserstein distance, *f*-divergences (e.g., the KL divergence) [52], and the maximum mean discrepancy [53]. If information about the target distribution is available, it can be incorportated in the definition of the uncertainty set. An approach related to robust optimization is to strive to minimize not only the empirical loss *L*(*Y, f*(*X*)) but also its variance [54, 55].

It is also useful to assess model performance across values of demographic variables such as age, socioeconomic status, or ethnicity. Indeed, a good overall prediction performance can be achieved despite a poor performance on a minority group. Ensuring that a predictor performs well for all subpopulations reduces sensitivity to potential shifts in demographics and is essential to ensure fairness [35]. For instance, there is a risk that machine-learning analysis of dermoscopic images under-diagnoses malignant moles on skin tones that are typically under-represented in the training set [56]. Fairness is especially relevant when the model output could be used to grant access to some treatment. Because similar issues arise in many applications of machine learning, there is a growing literature on fairness (see, e.g., [34], for an overview). For instance, Duchi and Namkoong [52] show that distributionally robust optimization can improve performance on under-represented subpopulations.

### Multi-site datasets

Often datasets are collected across several sites or hospitals, or with different measurement devices. This heterogeneity provides an opportunity to train models that generalize to unseen sites or devices. Some studies attempt to remove site effects by regressing all features on the site indicator variable. For the same reasons that regressing out age is detrimental in Fig. 1, this strategy often gives worse generalization across sites.

Data harmonization, such as compensating differences across measurement devices, is crucial but remains difficult and cannot correct these differences perfectly [57]. Removing too much intersite variance can lead to loss of informative signal. Rather, it is important to model it well, accounting for the 2 sources of variance, across participants and across sites. A good model strives to yield good results on all sites. One solution is to adapt ideas from robust optimization: on data drawn from different distributions (e.g., from several sites), Krueger et al. [58] show the benefits of minimizing the empirical risk on the worse site or adding penalties on the variance of the loss across sites.

Measures of prediction performance should aggregate scores at the site level (not pooling all individuals) and check the variance across sites and the performance on the worse site. Cross-validation schemes should hold out entire sites [12, 59].

## Special Cases of Dataset Shift

Categorizing dataset shift helps in finding the best approach to tackle it [27, 60]. We summarize 2 frequently met scenarios that are easier to handle than the general case and can call for different adjustments: covariate shift and prior probability shift.

### Covariate shift

**Figure 5:**
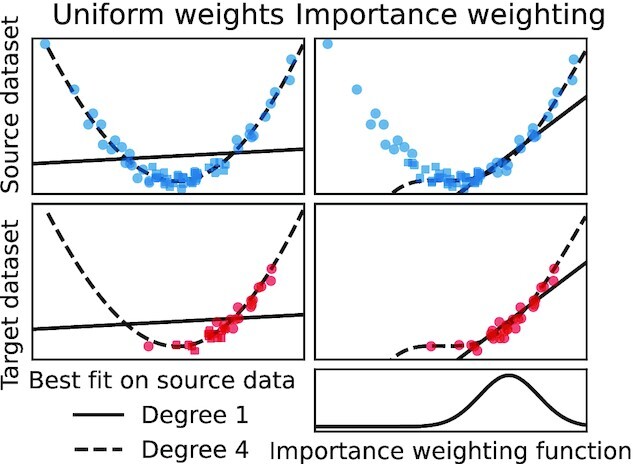
Covariate shift: $P (Y \, |\, X)$ stays the same, but the feature space is sampled differently in the source and target datasets. A powerful learner may generalize well as $P (Y \, |\, X)$ is correctly captured [27]. Thus the polynomial fit of degree 4 performs well on the new dataset. However, an overconstrained learner such as the linear fit can benefit from reweighting training examples to give more importance to the most relevant region of the feature space.

Covariate shift occurs when the marginal distribution of *X* changes between the source and target datasets [i.e., *p_t_*(*x*) ≠ *p_s_*(*x*)] but $P(Y \, |\, X)$ stays the same. This happens, e.g., in the second scenario in Fig. 3, where sample selection based on *X* (but not *Y*) changes the distribution of the inputs. If the model is correctly specified, an estimator trained with uniform weights will lead to optimal predictions given sufficient training data (prediction consistency [61], Lemma 4). However the usual (unweighted) estimator is not consistent for an over-constrained (misspecified) model. Indeed, an over-constrained model may be able to fit the data well only in some regions of the input feature space (Fig. 1). In this case reweighting training examples (Section “Importance weighting: a generic tool against dataset shift") to give more importance to those that are more representative of the target data is beneficial [27, 38]. Figure 5 illustrates covariate shift.

### Prior probability shift

**Figure 6:**
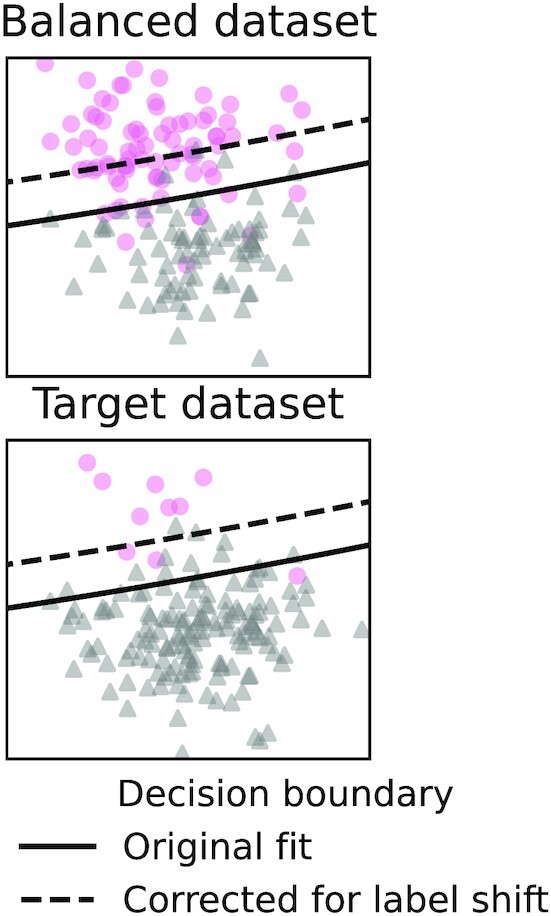
Prior probability shift: when *P*(*Y*) changes but $P(X \, |\, Y)$ stays the same. This can happen for example when participants are selected on the basis of *Y*—possibly to have a dataset with a balanced number of patients and healthy participants: $X \leftarrow Y \rightarrow \mathrm{\fbox{S}}$. When the prior probability (marginal distribution of *Y*) in the target population is known, this is easily corrected by applying Bayes’ rule. The output *Y* is typically low-dimensional and discrete (often it is a single binary value), so *P*(*Y*) can often be estimated precisely from few examples.

Another relatively simple case of dataset shift is “prior probability shift." With prior probability shift (aka label shift or target shift), the distribution of *Y* changes but not $P(X \, |\, Y)$. This happens for example when disease prevalence changes in the target population but manifests itself in the same way. Even more frequently, prior probability shift arises when 1 rare class is over-represented in the training data so that the dataset is more balanced, as when extracting a biomarker from a case-control cohort, or when the dataset is resampled as a strategy to handle the “class imbalance" problem [62]. Prior probability shift can be corrected without extracting a new biomarker, simply by adjusting a model’s predicted probabilities using Bayes’ rule (as noted, e.g., in [27, 38]). When the classes are well separated, the effect of this correction may be small; i.e.,  the uncorrected classifier may generalize well without correction. Fig. 6 illustrates prior probability shift.

## Conclusion

Ideally, machine-learning biomarkers would be designed and trained using datasets carefully collected to be representative of the targeted population—as in Liu et al. [63]. To be trusted, biomarkers ultimately need to be evaluated rigorously on 1 or several independent and representative samples. However, such data collection is expensive. It is therefore useful to exploit existing datasets in an opportunistic way as much as possible in the early stages of biomarker development. When doing so, correctly accounting for dataset shift can prevent wasting important resources on machine-learning predictors that have little chance of performing well outside of 1 particular dataset.

We gave an overview of importance weighting, a simple tool against dataset shift. Importance weighting needs a clear definition of the targeted population and access to a diverse training dataset. When this is not possible, distributionally robust optimization may be a promising alternative, although it is a more recent approach and more difficult to implement. Despite much work and progress, dataset shift remains a difficult problem. Characterizing its impact and the effectiveness of existing solutions for biomarker discovery will be important for machine-learning models to become more reliable in healthcare applications.

We conclude with the following recommendations:

- Be aware of the dataset shift problem and the difficulty of out-of-dataset generalization. Do not treat cross-validation scores on 1 dataset as a guarantee that a model will perform well on clinical data.
- Collect diverse, representative data.
- Use powerful machine-learning models and large datasets.
- Consider using importance weighting to correct biases in the data collection, especially if the learning model may be over-constrained (e.g., when using a linear model).
- Look for associations between prediction performance and demographic variables in the validation set to detect potential generalization or fairness issues.
- Do not remove “confounding signal" in a predictive setting.

These recommendations should help in designing fair biomarkers and their efficient application on new cohorts.

## Data Availability

The source files used to create this publication can be found in the suppording data in the GigaScience Database [64]. They are also available in a Git repository [65]. The UK Biobank data used in this study are Controlled Access Data. To access these data, one would need to visit the UKBiobank website [66] and follow the steps for registering, applying for access, and downloading the data. The data used in this study were the “tabular” dataset that is archived in UK Biobank (i.e., the data that are neither imaging nor genome sequencing). This research has been conducted using the UK Biobank Resource under Application No. 45551.

## Abbreviations

AUC: area under the curve; CT: computed tomographic; FEV1: Forced expiratory volume in 1 second; RBF: Radial Basis Function; SVM: Support Vector Machines.

## Competing Interests

The authors declare that they have no competing interests.

## Authors' Contributions

All authors participated in conception, literature search, data interpretation, and editing the manuscript. J.D. wrote the software and drafted the manuscript.

## Supplementary Material

giab055_GIGA-D-21-00081_Original_Submission

giab055_GIGA-D-21-00081_Revision_1

giab055_GIGA-D-21-00081_Revision_2

giab055_Response_to_Reviewer_Comments_Original_Submission

giab055_Response_to_Reviewer_Comments_Revision_1

giab055_Reviewer_1_Report_Original_Submission Guray Erus -- 5/10/2021 Reviewed

giab055_Reviewer_1_Report_Revision_1 Guray Erus -- 7/13/2021 Reviewed

giab055_Reviewer_2_Report_Original_Submission Spencer Thomas -- 5/13/2021 Reviewed

giab055_Reviewer_3_Report_Original_Submission Enzo Ferrante -- 5/15/2021 Reviewed

giab055_Reviewer_3_Report_Revision_1 Enzo Ferrante -- 7/18/2021 Reviewed

## Definition and Estimation of Importance Weights

We will implicitly assume that all the random variables we consider admit densities and denote *p_s_* and *p_t_* the density of the joint distribution of (*X, Y*) applied to the source and target populations, respectively. If the support of *p_t_* is included in that of *p_s_* (meaning that *p_s_* > 0 wherever *p_t_* > 0), we have:

(A1) \begin{eqnarray*}
\mathbb {E} _{\text{target}}[\, L(Y,\, f(X))\, ] = \mathbb {E} _{\text{source}}\left[\, \frac{p_t(X,\, Y)}{p_s(X,\, Y)}\,\,L(Y,\, f(X))\, \right] , \end{eqnarray*}

where *L* is the cost function and *f* is a prediction function, $\mathbb {E} _{\text{source}}$ (respectively, $\mathbb {E} _{\text{target}}$) the expectation on the source (respectively, target) data. The risk (on target data) can therefore be computed as an expectation on the source distribution where the loss function is reweighted by the importance weights:

(A2) \begin{eqnarray*}
w(x,y) = \frac{p_t(x,y)}{p_s(x,y)}. \end{eqnarray*}

If $\hat{w}$ are empirical estimates of the importance weights *w*, it is possible to compute the reweighted empirical risk:

(A3) \begin{eqnarray*}
\hat{R}_{\hat{w}}(f) = \sum \nolimits_{i=1}^n \hat{w}(x_i, y_i)\, L(y_i, f(x_i)). \end{eqnarray*}

Rather than being weighted, examples can also be resampled with importance or rejection sampling [67, 68]. Importances can also be taken into account for model selection; e.g., in Sugiyama et al. [69] examples of the test set are also reweighted when computing cross-validation scores. Cortes et al. [70] study how errors in the estimation of the weights affect the prediction performance.

### Preferential sample selection and inverse probability weighting

In the case of preferential sample selection (Section "Preferential sample selection: a common source of shift"), the condition that requires for the support of *p_t_* to be included in the support of *p_s_* translates to a requirement that all individuals have a non-zero probability of being selected: $P(S=1 \, |\, x,y) > 0$ for all (*x, y*) in the support of *p_t_*. When this is verified, by applying Bayes’ rule the definition of importance weights in Equation (A2) can be reformulated (see [70], Sec. 2.3):

(A4) \begin{eqnarray*}
w(x, y) = \frac{P(S=1)}{P(S=1 \, |\, X=x,Y=y)}. \,\, \end{eqnarray*}

These weights are sometimes called inverse probability weights [71] or inverse propensity scores [72]. Training examples that had a low probability of being selected receive higher weights because they have to account for similar individuals who were not selected.

### Computing importance weights

In practice *p_t_*(*x, y*), which is the joint density of (*X, Y*) in the target data, is not known. However, it is not needed for the estimation of *p_t_*/*p_s_*. More efficient estimation hinges on 2 observations: estimation of both densities separately is not necessary to estimate their ratio, and variables that have the same distribution in source and target data can be factored out.

Here we describe methods that estimate the true importance weights *p_t_*/*p_s_*, but we point out that reweighting the training examples reduces the bias of the empirical risk but increases the variance of the estimated model parameters. Even when the importances are perfectly known, it can therefore be beneficial to regularize the weights [61].

#### Computing importance weights does not require estimation of distribution densities

Importance weights can be computed by modeling *p_s_* and *p_t_* separately and then computing their ratio ([73], Sec. 4.1). However, distribution density estimation is notoriously difficult; non-parametric methods are hindered by the curse of dimensionality and parametric methods depend heavily on the correct specification of a parametric form.

But estimating both densities is more information than is needed to compute the sample weights. Instead, one can directly optimize importance weights to make the reweighted sample similar to the target distribution, by matching moments [74] or mean embeddings [75, 76], minimizing the KL-divergence [77], solving a least-squares estimation problem [78], or with optimal transport [49].

Alternatively, a discriminative model can be trained to distinguish source and target examples. In the specific case of preferential sample selection, this means directly estimating the probability of selection *P*(*S* = 1) (cf. Equation A4). In general, the shift is not always due to selection: the source data are not necessarily obtained by subsampling the target population. In this case we denote *T* = 1 if an individual comes from the target data and *T* = 0 if it comes from the source data. Then, a classifier can be trained to predict from which dataset (source or target) a sample is drawn, and the importance weights obtained from the predicted probabilities ([73], Sec. 4.3):

(A5) \begin{eqnarray*}
w(x, y) = \frac{P(T=1 \, |\, X=x, Y=y)\, P(T=0)}{P(T=0 \, |\, X=x, Y=y)\, P(T=1)}. \end{eqnarray*}

The classifier must be calibrated (i.e., produce accurate probability estimates, not only a correct decision; see Niculescu-Mizil and Caruana [79]). Note that constant factors such as *P*(*T* = 0)/*P*(*T* = 1) usually do not matter and are easy to estimate if needed. This discriminative approach is effective because the distribution of $(T \, |\, X=x, Y=y)$ is much easier to estimate than the distribution of $(X, Y \, |\, T=t)$ : *T* is a single binary variable whereas (*X, Y*) is high-dimensional and often continuous.

The classifier does not need to distinguish source and target examples with high accuracy. In the ideal situation of no dataset shift, the classifier will perform at chance level. On the contrary, a high accuracy means that there is little overlap between the source and target distributions and the model will probably not generalize well.

#### What distributions differ in source and target data?

When computing importance weights, it is possible to exploit prior knowledge that some distributions are left unchanged in the target data. For example,

(A6) \begin{eqnarray*}
\frac{p_t(x, y)}{p_s(x, y)} = \frac{p_t(y \, |\, x)\, p_t(x)}{p_s(y \, |\, x)\, p_s(x)}. \end{eqnarray*}

Imagine that the marginal distribution of input *X* differs in source and target data, but the conditional distribution of the output *Y* given the input stays the same: *p_t_*(*x*) ≠ *p_s_*(*x*) but $p_t(y \, |\, x) = p_s(y \, |\, x)$ (a setting known as “covariate shift"). Then, the importance weights simplify to

(A7) \begin{eqnarray*}
w(x, y) = \frac{p_t(x)}{p_s(x)}. \end{eqnarray*}

In this case, importance weights can be estimated using only unlabeled examples (individuals for whom *Y* is unknown) from the target distribution.

Often, the variables that influence selection (e.g., demographic variables such as age) are lower-dimensional than the full features (e.g., high-dimensional images), and dataset shift can be corrected with limited information on the target distribution, with importance weights or otherwise. Moreover, even if additional information *Z* that predicts selection but is independent of (*X, Y*) is available, it should not be used to compute the importance weights. Indeed, this would only increase the weights’ variance without reducing the bias due to the dataset shift ([21], Sec. 15.5).

## Tobacco smoking prediction in the UKBiobank

We consider predicting the smoking status of participants in the UKBiobank study to illustrate the effect of dataset shift on prediction performance. Smoking is associated with neuropathologies in patients with schizophrenia, mood or anxiety disorders, substance use and other brain related disorders.

A total of 6,000 participants are used in a preliminary step to identify the 29 most relevant predictive features (listed in Appendix B.1), by cross-validating a gradient boosting model and computing permutation feature importances. We then draw 2 samples of 100,000 individuals from the rest of the dataset that have different age distributions. In the young sample, 90% of individuals come from the youngest 20% of the dataset, and the remaining 10% are sampled from the oldest 20% of the dataset. In the old sample, these proportions are reversed. We then perform 10-fold cross-validation. For each fold, both the training and testing set can be drawn from either the young or the old population, resulting in 4 tasks on which several machine-learning estimators are evaluated. We use this experiment to compare 2 machine-learning models: a simple one—regularized linear support vector classifier, and a flexible one—gradient boosting. For each classifier, 3 strategies are considered to handle the dataset shift: (i) baseline—the generic algorithm without modifications, (ii) importance weighting (Section “Importance weighting: a generic tool against dataset shift"), and (iii) the (unfortunately popular) non-solution: “regressing out the confounder”—regressing the predictive features on the age and using the residuals as inputs to the classifier.

The results are similar to those seen with simulated data in Fig. 1. For a given learner and test population, training on a different population degrades the prediction score. For example, if the learner is to be tested on the young population, it performs best when trained on the young population. Gradient boosting vastly outperforms the linear model in all configurations. Regressing out the age always degrades the prediction; it is always worse than the unmodified baseline, whether a dataset shift is present or not. Finally, importance weighting improves the predictions of the over-constrained (misspecified) linear model in the presence of dataset shift but degrades the prediction of the powerful learner used in this experiment. This is due to the fact that the gradient boosting already captures the correct separation for both young and old individuals, and therefore importance weighting does not bring any benefit but only reduces the effective training sample size by increasing the variance of the empirical risk.

### Features used for tobacco smoking status prediction

The 30 most important features were identified in a preliminary experiment with 6,000 participants (that were not used in the subsequent analysis). One of these features, “Date F17 first reported (mental and behavioural disorders due to use of tobacco),” was deemed trivial—too informative because it directly implies that the participant does smoke tobacco, and removed. The remaining 29 features were used for the experiment described in Section “False solutions to tackling dataset shift".

- Forced expiratory volume in 1 second (FEV1), predicted percentage
- Lifetime number of sexual partners
- Age first had sexual intercourse
- Age when last used cannabis
- Ever used cannabis
- FEV1, predicted
- Acceptability of each blow result
- Mouth/teeth dental problems
- Coffee intake
- FEV1/ Forced vital capacity ratio Z-score
- Alcohol intake frequency
- Date J44 first reported (other chronic obstructive pulmonary disease)
- Former alcohol drinker
- Average weekly spirits intake
- Year of birth
- Acceptability of each blow result
- Date of chronic obstructive pulmonary disease report
- Leisure/social activities
- Morning/evening person (chronotype)
- Mean sphered cell volume
- Lymphocyte count
- Townsend deprivation index at recruitment
- Age hay fever, rhinitis, or eczema diagnosed
- Age started oral contraceptive pill use
- White blood cell (leukocyte) count
- Age completed full-time education
- Age at recruitment
- Workplace had a lot of cigarette smoke from other people smoking
- Wheeze or whistling in the chest in past year

## Glossary

Here we provide a summary of some terms and notations used in the article.

Target population: the population on which the biomarker (machine-learning model) will be applied.
Source population: the population from which the sample used to train the machine-learning model is drawn.
Selection: in the case that source data are drawn (with non-uniform probabilities) from the target population, we denote by *S* = 1 the fact that an individual is selected to enter the source data (e.g.,  to participate in a medical study).
Provenance of an individual: when samples from both the source and the target populations (e.g., Appendix A.2.1) are available, we also denote *T* = 1 if an individual comes from the target population and *T* = 0 if they come from the source population.
Confounding: in “causal inference," when estimating the effect of a treatment on an outcome, confounding occurs if a third variable (e.g., age, a comorbidity, the seriousness of a condition) influences both the treatment and the outcome, possibly producing a spurious statistical association between the two. This notion is not directly relevant to dataset shift, and we mention it only to insist that it is a different problem. See Hernán and Robins [21], Chap. 7, for a more precise definition.
Domain adaptation: the task of designing machine-learning methods that are resilient to dataset shift—essentially a synonym for dataset shift, i.e.,  another useful search term for readers looking for further information on this problem.
