# Supplementary material for: Preventing dataset shift from breaking machine-learning biomarkers
Source: Gigascience. 2021 Sep 28;10(9):giab055. doi: 10.1093/gigascience/giab055 (PMC8478611; doi:10.1093/gigascience/giab055)
Supplement: giab055_GIGA-D-21-00081_Original_Submission [file giab055_giga-d-21-00081_original_submission.pdf]

GigaScience

# Preventing dataset shift from breaking machine-learning biomarkers

--Manuscript Draft--

| | |
|---|---|
| **Manuscript Number:** | GIGA-D-21-00081 |
| **Full Title:** | Preventing dataset shift from breaking machine-learning biomarkers |
| **Article Type:** | Review |
| **Funding Information:** | National Institutes of Health (NIH-NIBIB P41 EB019936) — Dr Jean-Baptiste Poline<br>National Institute of Mental Health (NIH-NIMH R01 MH083320) — Dr Jean-Baptiste Poline<br>National Institutes of Health (NIH RF1 MH120021) — Dr Jean-Baptiste Poline<br>National Institute of Mental Health (R01MH096906) — Dr Jean-Baptiste Poline |
| **Abstract:** | Machine learning brings the hope of finding new biomarkers built from cohorts with rich biomedical measurements. A good biomarker is one that gives reliable detection of the corresponding condition. However, biomarkers are often extracted from a cohort that differs from the target population. Such a mismatch, known as a dataset shift, can undermine the application of the biomarker to new individuals. Dataset shifts are frequent in biomedical research, for example because of recruitment biases. When a dataset shift occurs, standard machine-learning techniques do not suffice to extract and validate biomarkers. This article provides an overview of when and how dataset shifts break machine-learning extraction of biomarkers, as well as detection and correction strategies. |
| **Corresponding Author:** | Jérôme Dockès<br>McGill University<br>Montréal, CANADA |
| **Corresponding Author Secondary Information:** | |
| **Corresponding Author's Institution:** | McGill University |
| **Corresponding Author's Secondary Institution:** | |
| **First Author:** | Jérôme Dockès |
| **First Author Secondary Information:** | |
| **Order of Authors:** | Jérôme Dockès<br>Gaël Varoquaux<br>Jean-Baptiste Poline |
| **Order of Authors Secondary Information:** | |
| **Additional Information:** | |
| **Question** | **Response** |
| Are you submitting this manuscript to a special series or article collection? | No |
| **Experimental design and statistics**<br><br>Full details of the experimental design and statistical methods used should be given in the Methods section, as detailed in our | Yes |

Powered by Editorial Manager® and ProduXion Manager® from Aries Systems Corporation

| | |
|---|---|
| Minimum Standards Reporting Checklist. Information essential to interpreting the data presented should be made available in the figure legends.<br><br>Have you included all the information requested in your manuscript? | |
| **Resources**<br><br>A description of all resources used, including antibodies, cell lines, animals and software tools, with enough information to allow them to be uniquely identified, should be included in the Methods section. Authors are strongly encouraged to cite Research Resource Identifiers (RRIDs) for antibodies, model organisms and tools, where possible.<br><br>Have you included the information requested as detailed in our Minimum Standards Reporting Checklist? | Yes |
| **Availability of data and materials**<br><br>All datasets and code on which the conclusions of the paper rely must be either included in your submission or deposited in publicly available repositories (where available and ethically appropriate), referencing such data using a unique identifier in the references and in the “Availability of Data and Materials” section of your manuscript.<br><br>Have you have met the above requirement as detailed in our Minimum Standards Reporting Checklist? | Yes |

Manuscript Click here to access/download;Manuscript;main.pdf

OXFORD
(GIGA)$^n$ SCIENCE

*GigaScience*, 20xx, 1–12

doi: xx.xxxx/xxxx
**Manuscript in Preparation**
**Paper**

PAPER

# Preventing dataset shift from breaking machine-learning biomarkers

Jérôme Dockès[1, *], Gaël Varoquaux[1, 2,†] and Jean-Baptiste Poline[1, †]

[1]McGill University and [2]INRIA

[*]Corresponding author.
[†]JB Poline and Gaël Varoquaux contributed equally to this work.

## Abstract

Machine learning brings the hope of finding new biomarkers built from cohorts with rich biomedical measurements. A good biomarker is one that gives reliable detection of the corresponding condition. However, biomarkers are often extracted from a cohort that differs from the target population. Such a mismatch, known as a dataset shift, can undermine the application of the biomarker to new individuals. Dataset shifts are frequent in biomedical research, e.g. because of recruitment biases. When a dataset shift occurs, standard machine-learning techniques do not suffice to extract and validate biomarkers. This article provides an overview of when and how dataset shifts break machine-learning extraction of biomarkers, as well as detection and correction strategies.

## 1 Introduction: dataset shift breaks learned biomarkers

Biomarkers are measurements that provide information about a medical condition or physiological state [1]. For example, the presence of an antibody may indicate an infection; a complex combination of features extracted from a medical image can help assess the evolution of a tumor. Biomarkers are important for diagnosis, prognosis, and treatment or risk assessments.

Complex biomedical measures may carry precious medical information, as with histopathological images or genome sequencing of biopsy samples in oncology. Building quantitative biomarkers from these requires sophisticated statistical analysis. With large datasets becoming accessible, supervised machine learning provides new promises as it can optimize the information extracted to relate to a specific output variable of interest,

**Compiled on:** March 9, 2021.
Draft manuscript prepared by the author.

such as a cancer diagnosis [2, 3, 4]. These methods, cornerstones of artificial intelligence, are starting to appear in clinical practice: a machine-learning based radiological tool for breast-cancer diagnosis has recently been approved by the FDA[1].

Can such biomarkers, built from complex data processing, be safely used in clinical practice, beyond the initial research settings? One risk is that there can be a mismatch, or *dataset shift*, between the distribution of the individuals used to estimate this statistical link and that of the target population that should benefit from the biomarker. In this case, the extracted associations may not apply to the target population [5]. Computer aided diagnostic of thoracic diseases from X-ray images has indeed been shown to be unreliable for individuals of a given sex if built from a cohort over-representing the other sex [6]. More generally, biomarkers may fail on data from different imaging devices, hospitals, populations with a different age distribution, *etc.*. Dataset biases are frequent in medicine. For instance selection biases –*eg* due to volunteering self-selection, non-response, dropout...– [7, 8] may cause cohorts to capture only a small range of possible patients and disease manifestations in the presence of spectrum effects [9, 10]. Dataset shift or dataset bias can cause systematic errors that cannot be fixed by acquiring larger datasets and require specific methodological care.

In this article, we consider biomarkers built with supervised machine learning. We characterize the problem of dataset shift, show how it can hinder the use of machine learning for health applications [11, 12], and provide mitigation strategies.

## 2 A primer on machine learning for biomarkers

### 2.1 Empirical Risk Minimization

Let us first introduce the principles of machine learning used to build biomarkers. Supervised learning captures from observed data the link between a set of input measures (features) $X$ and an output (e.g. a condition) $Y$: for example the relation between the absorption spectrum of oral mucosa and blood glucose concentration [13]. A supervised learning algorithm finds a function $f$ such that $f(X)$ is as close as possible to the output $Y$. Following machine-learning terminology, we call the system's best guess $f(x)$ for a value $x$ a *prediction*, even when it does not concern a measurement in the future.

Empirical Risk Minimization, central to machine learning, uses a loss function $L$ to measure how far a prediction $f(x)$ is from the true value $y$, for example the squared difference:

$$L(y, f(x)) = (y - f(x))^2 \; . \qquad (1)$$

The goal is to find a function $f$ that has a small *risk*, which is the *expected* loss on the true distribution of $X$ and $Y$, i.e. on *unseen individuals*. The true risk cannot be computed in practice: it would require having seen all possible patients, the true distribution of patients. The *empirical* risk is used instead: the average error over available examples,

$$\hat{R}(f) = \frac{1}{n}\sum_{i=1}^{n} L(y_i, f(x_i)) \; , \qquad (2)$$

where $\{(x_i, y_i)\,,\; i = 1, \ldots, n\}$ are available $(X, Y)$ data, called *training* examples. The statistical link of interest is then approximated by choosing $f$ within a family of candidate functions as the one that minimizes the empirical risk $\hat{R}(f)$.

The crucial assumption underlying this very popular approach is that the biomarker $f$ will then be applied to individuals drawn from the same population as the training examples $\{x_i, y_i\}$. It can be important to distinguish the *source* data, used to fit and evaluate a biomarker (e.g. a dataset collected for research), from the *target* data, on which the biomarker is meant to be used for clinical applications (e.g. new visitors of a hospital). Indeed, if the training examples are not representative of the target population – if there is a dataset shift – the empirical risk is a poor estimate of the expected error, and $f$ will not perform well on individuals from the target population.

### 2.2 Evaluation: Independent test set and cross-validation

Once a biomarker has been estimated from training examples, measuring its error on these same individuals results in an optimistic estimate of the risk, the expected error on un-

[1] https://fda.report/PMN/K192854

seen individuals [14, 15, Sec. 7.4]. To obtain valid estimates of the expected performance on new data, the error is measured on an independent sample held out during training, called the test set. The most common approach to obtain such a test set is to randomly split the available data. This process is usually repeated with several splits, a procedure called cross-validation [16, 15, Sec. 7].

When training and test examples are chosen uniformly from the same sample, they are drawn from the same distribution (i.e. the same population): there is no dataset shift. Some studies also measure the error on an *independent* dataset [e.g. 17, 18]. This helps establishing external validity, assessing whether the predictor will perform well outside of the dataset used to define it [19]. Unfortunately, the biases in participant recruitment may be similar in independently collected datasets. For example if patients with severe symptoms are difficult to recruit, this is likely to distort all datasets similarly. Testing on a dataset collected independently is therefore a useful check, but no silver bullet to rule out dataset shift issues.

## 3 Common misconceptions on tackling dataset shift

We now point out some misconceptions and confusions with problems not directly related to dataset shift.

*Dataset shift differs from confounding..* The machine-learning methods we consider here capture statistical associations, but *do not target causal effects*. For biomarkers, the association itself is interesting, whether causal or not. Elevated body temperature may be the consequence of a condition, but also cause a disorder. It is a clinically useful measure in both settings. The notion of confounding is one of *causal analysis*, and does not relate to *predictive analysis*, as pointed out by seminal textbooks: "if the goal of the data analysis is purely predictive, no adjustment for confounding is necessary [...] the concept of confounding does not even apply."[20, Sec. 18.1], or Pearl [21]. In prediction settings, applying procedures meant to adjust for confounding generally degrades prediction performance without solving the dataset shift issue, as seen in Figure 1.

*Training examples should not be selected to be homogeneous..* To obtain valid predictive models that perform well beyond the training sample, it is crucial to collect datasets that represent the whole population and reflect its diversity as much as possible [5, 23, 24]. Yet clinical research often emphasizes the opposite: very homogeneous datasets and carefully selected participants. While this may help reduce variance and improve statistical testing, it degrades prediction performance and fairness.

*Simpler models are not less sensitive to dataset shift..* Often, flexible models can be more robust to dataset shifts, and thus generalize better, than linear models [25], as seen in Figures 1 and 5. Indeed, an over-constrained (ill-specified) model may only fit well a restricted region of the feature space, and its performance can degrade if the distribution of inputs changes, even if the relation to the output stays the same (i.e. when covariate shift occurs, Section 6.1).

Dataset shift does not call for simpler models as it is not a small-sample issue. Collecting more data will not correct systematic dataset bias.

## 4 Preferential sample selection: a common source of shift

In 2017, competitors in the million-dollar-prize data science bowl used machine learning to predict if individuals would be diagnosed with lung cancer within one year, based on a CT scan. Assuming that the winning model achieves satisfying accuracy on left-out examples from this dataset, is it ready to be deployed in hospitals? Most likely not. Selection criteria may make this dataset not representative of the potential lung cancer patients general population. Selected participants verified many criteria, including being a smoker and not having recent medical problems such as pneumonia. How would the winning predictor perform on a more diverse population? For example, another disease could present features that the classifier could mistakenly take for signs of lung cancer. Beyond explicit selection criteria, many factors such as age, ethnicity, or socioeconomic status influence participation in biomedical studies [26, 27, 22, 28]. Not only can these shifts reduce overall predictive performance, they can also lead to discriminative

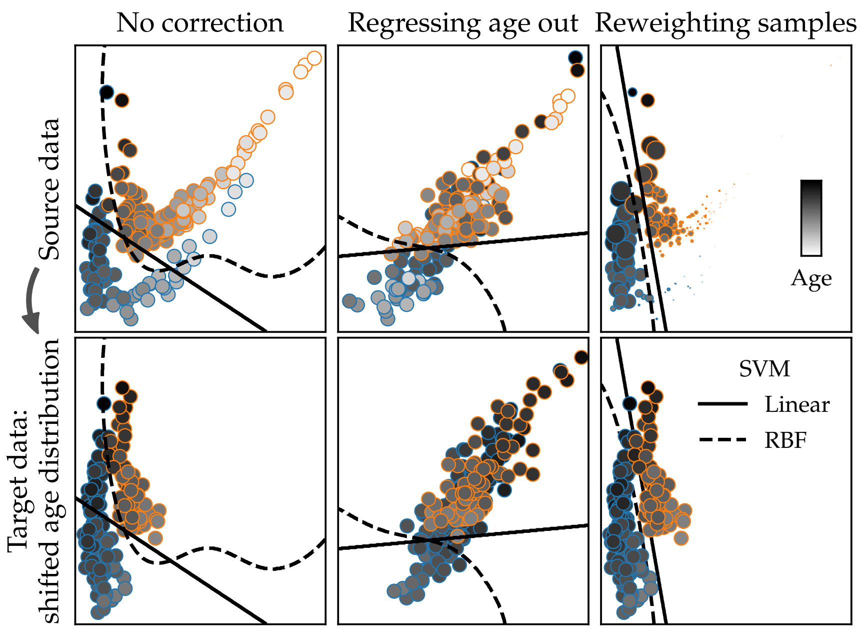


**Figure 1. Classification with dataset shift – regressing out a correlate of the shift does not help generalization.** We learn to classify patients (blue circles) from healthy subjects (orange circles), using 2-dimensional features. Age, indicated by color, influences both the features and the probability of disease (fig. 2). In a second dataset (bottom row), the process generating the data is the same but the age distribution is shifted: subjects tend to be older. This situation is often met in practice as the elderly are less likely to participate in clinical studies [22]. **First column:** no correction is applied. As the situation is close to a covariate shift (Section 6.1), a powerful learner (RBF-SVM) generalizes well to the second dataset. A misspecified model – Linear-SVM – generalizes poorly. **Second column:** wrong approach. To remove associations with age, features are replaced by the residuals after regressing them on age. This destroys the signal and results in poor performance for both models and datasets. **Third column:** Features are not modified but samples are weighted to give more importance to those that are more likely in the target distribution. Small circles indicate younger subjects, with less influence on the classifier estimation. This reweighting yields a better prediction for the older population.

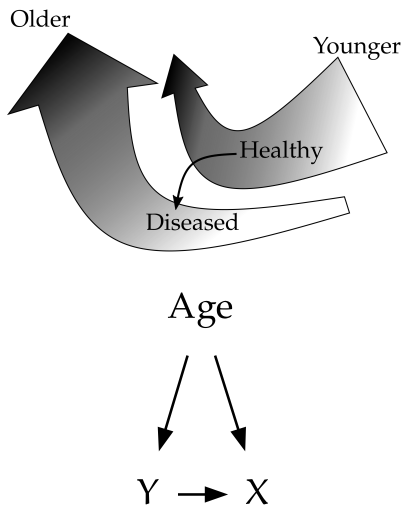


$$Y := g(\text{Age}) \tag{3}$$
$$X := h(\text{Age}, Y) \tag{4}$$

For some $g, h$.

**Figure 2. Generative process for data in Figure 1.** Age influences both the target $Y$ and the features $X$, and $Y$ also has an effect on $X$. Between the source and target datasets, the distribution of age changes.

clinical decisions for poorly represented populations [29, 30, 31, 32, 33].

The examples above are instances of preferential selection, which happens when members of the population of interest do not have equal probabilities of being included in the source dataset: the selection $S$ is not independent of $(X, Y)$. Preferential sample selection is ubiquitous and cannot always be prevented by careful study design [34]. It is therefore a major challenge to the construction of reliable and fair biomarkers. Beyond preferential sample selection, there are many other sources of dataset shifts, e.g. population changes over time or interventions such as the introduction of new diagnostic codes in Electronic Health Records [35].

### 4.1 The selection mechanism influences the type of dataset shift

The correction for a dataset shift depends on the nature of this shift, characterized by which and how distributions are modified [25]. Knowledge of the mechanism producing the dataset shift helps formulate hypotheses about

distributions that remain unchanged in the target data [36, 37, Chap. 5].

Figure 3 illustrates this process with a simulated example of preferential sample selection. We consider the problem of predicting the volume $Y$ of a tumor from features $X$ extracted from contrast CT images. These features can be influenced not only by the tumor size, but also by the dosage of a contrast agent $M$. The first panel of Figure 3 shows a selection of data independent of the image and tumor volume: there is no dataset shift. In the second panel, selection depends on the CT image itself (for example images with a low signal-to-noise ratio are discarded). As selection is independent of the tumor volume $Y$ given the image $X$, the distribution of images changes but the conditional distribution $P(Y \mid X)$ stays the same: we face a *covariate shift* (Section 6.1). The learned association remains valid. Moreover, reweighting examples to give more importance to those less likely to be selected can improve biomarkers for a target data (Section 5), and it can be done with only *unlabelled* examples from the target data. In the third panel, subjects who received a low contrast agent dose are less likely to enter the training dataset. Selection is therefore not independent of tumor volume (the output) given the image values (the input features). Therefore we have sample selection bias: the relation $P(Y \mid X)$ is different in source and target data, which will affect the performance of the prediction.

As these examples illustrate, the causal structure of the data helps identify the type of dataset shift and what information is needed to correct it.

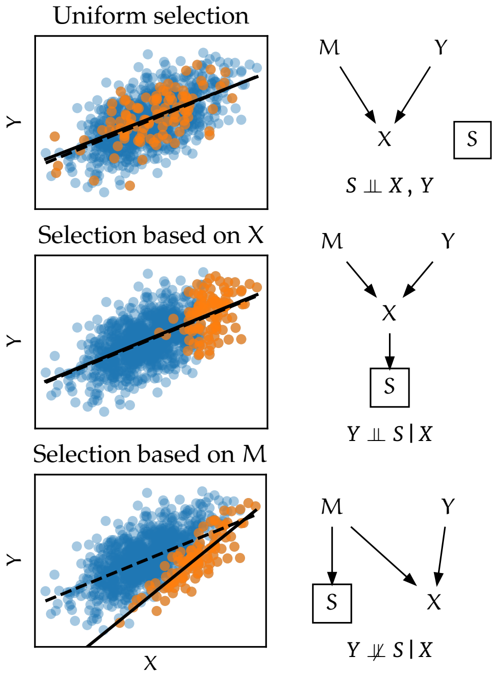


**Figure 3. Sample selection bias: three examples.** On the right are graphs giving conditional independence relations [38]. $Y$ is the lesion volume to predict (output). $M$ are the imaging parameters, e.g. contrast agent dosage. $X$ is the image, and depends both on $Y$ and $M$ (in this toy example $X$ is computed as $X := Y + M + \epsilon$, where $\epsilon$ is additive noise. $S$ indicates that data is selected to enter the source dataset (orange points) or not (blue points). The symbol $\perp\!\!\!\perp$ means independence between variables. Preferentially selecting samples results in a dataset shift (middle and bottom row). Depending on whether $Y \perp\!\!\!\perp S \mid X$, the conditional distribution of $Y \mid X$ – lesion volume given the image – estimated on the selected data may be biased or not.

## 5 Importance weighting: a generic tool against dataset shift

We now describe a solution to dataset shift that applies to many situations and can be easy to implement. We will not detail other approaches (e.g. invariant representations [39], data augmentation, adversarial methods), because they require implementing new learning algorithms or only apply to specific situations. Weiss et al. [40] and Pan and Yang [41] give systematic reviews of transfer learning.

Dataset shift occurs when the joint distribution of the features and outputs is different in the source (data used to fit the biomarker)

and in the target data. Informally, importance weighting consists in *reweighting or resampling* the available data to create a pseudo-sample that follows the same distribution as the target population.

To do so, examples are reweighted by their *importance weights* – the ratio of their likelihood in target data over source data. Examples that are rare in the source data but are likely in the target data are more relevant and therefore receive higher weights. Many statistical learning algorithms – including Support Vector Machines, decision trees, random forests, neural networks – naturally support weighting the training examples. Therefore, the challenge relies mostly in the estimation of the appropriate sample weights and the learning algorithm itself does not need to be modified.

To successfully use importance weighting, no part of the target distribution should be completely unseen. For example, if we use sex (among other features) to predict heart failure and our dataset only includes men, importance weighting cannot transform this dataset and make its sex distribution similar to that of the general population (Figure 4). Conversely, the source distribution may be broader than the target distribution (as seen for example in Figure 1).

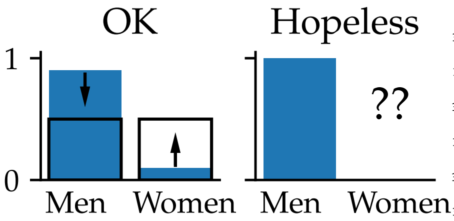


**Figure 4. Left:** distribution of sex can be balanced by downweighting men and upweighting women. **Right:** women are completely missing; the dataset shift cannot be fixed by importance weighting.

In Appendix A, we provide a more precise definition of the importance weights, as well as an overview of how they can be estimated and used.

## 6 Special cases of dataset shift

Storkey [25] and Moreno-Torres et al. [42] provide a comprehensive categorization of dataset shifts. We summarize two frequently-met scenarios that can call for different adjustments: covariate shift and prior probability shift.

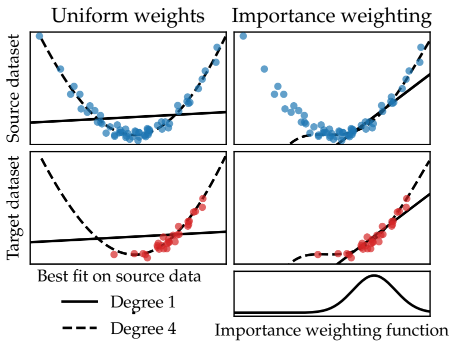


**Figure 5. Covariate shift:** $P(Y \mid X)$ stays the same but the feature space is sampled differently in the source and target datasets. A powerful learner may generalize well as $P(Y \mid X)$ is correctly captured [25]. Thus the polynomial fit of degree 4 performs well on the new dataset. However, an overconstrained learner such as the linear fit can benefit from reweighting training examples to give more importance to the most relevant region of the feature space.

### 6.1 Covariate shift

Covariate shift occurs when the marginal distribution of $X$ changes between the source and target datasets (i.e. $p_t(x) \neq p_s(x)$), but $P(Y \mid X)$ stays the same. This happens for example in the second scenario in Figure 3, where sample selection based on $X$ (but not $Y$) changes the distribution of the inputs. If the model is correctly specified, an estimator trained with uniform weights will lead to optimal predictions given sufficient training data [prediction consistency 43, Lemma 4]. However the usual (unweighted) estimator is not consistent for an over-constrained (misspecified) model. Indeed, a misspecified model may be able to fit the data well only in some regions of the input feature space (Figure 1). In this case reweighting training examples to give more importance to those that are more representative of the target data is beneficial [25, 36]. Figure 5 illustrates covariate shift.

### 6.2 Prior probability shift

With prior probability shift (a.k.a. label shift or target shift), the distribution of $Y$ changes but not $P(X \mid Y)$. This happens for example if one rare class is over-represented in the training data so that the dataset is more balanced, as when extracting a biomarker from a case-control cohort, or when disease prevalence changes in the target population but manifests itself in the same way. Prior probability

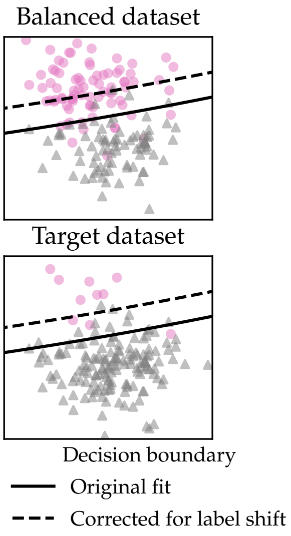


**Figure 6. Prior probability shift:** when $P(Y)$ changes but $P(X \mid Y)$ stays the same. This can happen for example when participants are selected based on $Y$ – possibly to have a dataset with a balanced number of patients and healthy participants: $X \leftarrow Y \rightarrow \boxed{S}$. When we know the prior probability (marginal distribution of $Y$) in the target population, this is easily corrected by applying Bayes' rule. The output $Y$ is typically low-dimensional and discrete (often it is a single binary value), so $P(Y)$ can often be estimated precisely from few examples.

shift can be corrected without extracting a new biomarker, simply by adjusting a model's predicted probabilities using Bayes' rule [as noted for example in 25, 36]. Figure 6 illustrates prior probability shift.

## 7 Conclusion

Ideally, machine learning biomarkers would be designed and trained using datasets carefully collected to be representative of the targeted population – as in Liu et al. [44]. To be trusted, the biomarker ultimately needs to be evaluated rigorously on an independent and representative sample. However, such data collection is expensive. It is therefore useful to exploit existing datasets in an opportunistic way as much as possible in the early stages of biomarker development. When doing so, correctly accounting for dataset shift can prevent wasting important resources on machine-learning predictors that have little chance of performing well outside of one particular dataset.

We gave an overview of importance weighting, an effective tool against dataset shift. Importance weighting needs a clear definition the targeted population and access to a diverse training dataset. When this is not possible, distributionally robust optimization is a promising alternative [see 45, for a review]. It consists in defining an ambiguity set – a set of distributions to which the target distribution might belong – then minimizing the worse risk across all distributions in this set. A related approach consists in ensuring the learner performs well for all inputs by penalizing the variance of the training error (loss) [46, 47]. These methods can help improve performance homogeneity across sub-populations and thus fairness [48, 49]. Even with distributionally robust optimization, a rich, diverse training set and any information about the target population remain extremely valuable. This technique is, to date, quite recent and more difficult to implement than importance weighting, as it requires adapting or designing new learning algorithms.

We conclude with some recommendations:

- collect diverse, representative data
- use importance weighting to correct biases in the data collection
- do not adjust for confounding in a predictive setting.

Following these recommendations should maximize building fair biomarkers and their efficient application on new cohorts.

*Author contributions.* Jérôme Dockès, Gaël Varoquaux and Jean-Baptiste Poline participated in conception, literature search, data interpretation, and editing the manuscript. Jérôme Dockès wrote the software and drafted the manuscript. Both Gaël Varoquaux and Jean-Baptiste Poline contributed equally to this work (as last authors).

*Competing interests statement.* The authors declare that there are no competing interests.

## References

1. Strimbu K, Tavel JA. What are biomarkers? Current Opinion in HIV and AIDS 2010;5(6):463.
2. Andreu-Perez J, Poon CC, Merrifield RD, Wong ST, Yang GZ. Big data for health. IEEE journal of biomedical and health informatics 2015;19(4):1193–1208.
3. Faust O, Hagiwara Y, Hong TJ, Lih OS, Acharya UR. Deep learning for healthcare applications based on physiological

signals: A review. Computer methods and programs in biomedicine 2018;161:1–13.

4. Deo RC. Machine learning in medicine. Circulation 2015;132(20):1920–1930.
5. Kakarmath S, Esteva A, Arnaout R, Harvey H, Kumar S, Muse E, et al. Best practices for authors of healthcare-related artificial intelligence manuscripts. NPJ Digital Medicine 2020;.
6. Larrazabal AJ, Nieto N, Peterson V, Milone DH, Ferrante E. Gender imbalance in medical imaging datasets produces biased classifiers for computer-aided diagnosis. Proceedings of the National Academy of Sciences 2020;117:12592.
7. Rothman KJ. Epidemiology: an introduction. Oxford university press; 2012.
8. Tripepi G, Jager KJ, Dekker FW, Zoccali C. Selection bias and information bias in clinical research. Nephron Clinical Practice 2010;115(2):c94–c99.
9. Ransohoff DF, Feinstein AR. Problems of spectrum and bias in evaluating the efficacy of diagnostic tests. New England Journal of Medicine 1978;299(17):926–930.
10. Mulherin SA, Miller WC. Spectrum bias or spectrum effect? Subgroup variation in diagnostic test evaluation. Annals of internal medicine 2002;137(7):598–602.
11. Woo CW, Chang LJ, Lindquist MA, Wager TD. Building better biomarkers: brain models in translational neuroimaging. Nature neuroscience 2017;20(3):365.
12. Wynants L, Van Calster B, Bonten MM, Collins GS, Debray TP, De Vos M, et al. Prediction models for diagnosis and prognosis of covid-19 infection: systematic review and critical appraisal. bmj 2020;369.
13. Kasahara R, Kino S, Soyama S, Matsuura Y. Noninvasive glucose monitoring using mid-infrared absorption spectroscopy based on a few wavenumbers. Biomedical optics express 2018;9(1):289–302.
14. Poldrack RA, Huckins G, Varoquaux G. Establishment of best practices for evidence for prediction: a review. JAMA psychiatry 2020;77(5):534–540.
15. Friedman J, Hastie T, Tibshirani R. The elements of statistical learning. Springer series in statistics New York; 2001.
16. Arlot S, Celisse A, et al. A survey of cross-validation procedures for model selection. Statistics surveys 2010;4:40–79.
17. Beck AH, Sangoi AR, Leung S, Marinelli RJ, Nielsen TO, Van De Vijver MJ, et al. Systematic analysis of breast cancer morphology uncovers stromal features associated with survival. Science translational medicine 2011;3(108):108ra113–108ra113.
18. Jin D, Zhou B, Han Y, Ren J, Han T, Liu B, et al. Generalizable, Reproducible, and Neuroscientifically Interpretable Imaging Biomarkers for Alzheimer's Disease. Advanced Science 2020;p. 2000675.
19. Bleeker S, Moll H, Steyerberg E, Donders A, Derksen-Lubsen G, Grobbee D, et al. External validation is necessary in prediction research:: A clinical example. Journal of clinical epidemiology 2003;56(9):826–832.
20. Hernán M, Robins J. Causal inference: What if. Boca Raton: Chapman & Hill/CRC 2020;.
21. Pearl J. The seven tools of causal inference, with reflections on machine learning. Communications of the ACM 2019;62(3):54–60.
22. Heiat A, Gross CP, Krumholz HM. Representation of the elderly, women, and minorities in heart failure clinical trials. Archives of internal medicine 2002;162(15).
23. England JR, Cheng PM. Artificial intelligence for medical image analysis: a guide for authors and reviewers. American Journal of Roentgenology 2019;212(3):513–519.
24. O'neil C. Weapons of math destruction: How big data increases inequality and threatens democracy. Broadway Books; 2016.
25. Storkey A. When training and test sets are different: characterizing learning transfer. Dataset shift in machine learning 2009;p. 3–28.
26. Henrich J, Heine SJ, Norenzayan A. Most people are not WEIRD. Nature 2010;466(7302):29–29.
27. Murthy VH, Krumholz HM, Gross CP. Participation in cancer clinical trials: race-, sex-, and age-based disparities. Jama 2004;291(22):2720–2726.
28. Chastain DB, Osae SP, Henao-Martínez AF, Franco-Paredes C, Chastain JS, Young HN. Racial disproportionality in Covid clinical trials. New England Journal of Medicine 2020;383(9):e59.
29. Oakden-Rayner L, Dunnmon J, Carneiro G, Ré C. Hidden stratification causes clinically meaningful failures in machine learn-

ing for medical imaging. In: Proceedings of the ACM Conference on Health, Inference, and Learning; 2020. p. 151–159.

30. Gianfrancesco MA, Tamang S, Yazdany J, Schmajuk G. Potential biases in machine learning algorithms using electronic health record data. JAMA internal medicine 2018;178(11):1544–1547.
31. Barocas S, Hardt M, Narayanan A. Fairness and Machine Learning. fairmlbook.org; 2019. http://www.fairmlbook.org.
32. Abbasi-Sureshjani S, Raumanns R, Michels BEJ, Schouten G, Cheplygina V. Risk of Training Diagnostic Algorithms on Data with Demographic Bias. In: Interpretable and Annotation-Efficient Learning for Medical Image Computing Cham: Springer International Publishing; 2020. p. 183–192.
33. Cirillo D, Catuara-Solarz S, Morey C, Guney E, Subirats L, Mellino S, et al. Sex and gender differences and biases in artificial intelligence for biomedicine and healthcare. NPJ Digital Medicine 2020;3(1):1–11.
34. Bareinboim E, Pearl J. Controlling selection bias in causal inference. In: Artificial Intelligence and Statistics; 2012. p. 100–108.
35. Sáez C, Gutiérrez-Sacristán A, Kohane I, García-Gómez JM, Avillach P. EHRtemporalVariability: delineating temporal dataset shifts in electronic health records. medRxiv 2020;.
36. Schölkopf B, Janzing D, Peters J, Sgouritsa E, Zhang K, Mooij J. On causal and anticausal learning. In: 29th International Conference on Machine Learning (ICML 2012) International Machine Learning Society; 2012. p. 1255–1262.
37. Peters J, Janzing D, Schölkopf B. Elements of causal inference: foundations and learning algorithms. MIT press; 2017.
38. Pearl J, Glymour M, Jewell NP. Causal inference in statistics: A primer. John Wiley & Sons; 2016.
39. Achille A, Soatto S. Emergence of invariance and disentanglement in deep representations. The Journal of Machine Learning Research 2018;19(1):1947–1980.
40. Weiss K, Khoshgoftaar TM, Wang D. A survey of transfer learning. Journal of Big data 2016;3(1):9.
41. Pan SJ, Yang Q. A survey on transfer learning. IEEE Transactions on knowledge and data engineering 2009;22(10):1345–1359.
42. Moreno-Torres JG, Raeder T, Alaiz-RodríGuez R, Chawla NV, Herrera F. A unifying view on dataset shift in classification. Pattern recognition 2012;45(1):521–530.
43. Shimodaira H. Improving predictive inference under covariate shift by weighting the log-likelihood function. Journal of statistical planning and inference 2000;90(2):227–244.
44. Liu M, Oxnard G, Klein E, Swanton C, Seiden M, Liu MC, et al. Sensitive and specific multi-cancer detection and localization using methylation signatures in cell-free DNA. Annals of Oncology 2020;.
45. Rahimian H, Mehrotra S. Distributionally robust optimization: A review. arXiv preprint arXiv:190805659 2019;.
46. Maurer A, Pontil M. Empirical Bernstein Bounds and Sample Variance Penalization. stat 2009;1050:21.
47. Namkoong H, Duchi JC. Variance-based regularization with convex objectives. In: Advances in neural information processing systems; 2017. p. 2971–2980.
48. Hashimoto T, Srivastava M, Namkoong H, Liang P. Fairness Without Demographics in Repeated Loss Minimization. In: International Conference on Machine Learning; 2018. p. 1929–1938.
49. Duchi J, Hashimoto T, Namkoong H. Distributionally Robust Losses for Latent Covariate Mixtures. arXiv preprint arXiv:200713982 2020;.
50. Zadrozny B, Langford J, Abe N. Cost-sensitive learning by cost-proportionate example weighting. In: Third IEEE international conference on data mining IEEE; 2003. p. 435–442.
51. Zadrozny B. Learning and evaluating classifiers under sample selection bias. In: Proceedings of the twenty-first international conference on Machine learning; 2004. p. 114.
52. Sugiyama M, Krauledat M, Müller KR. Covariate shift adaptation by importance weighted cross validation. Journal of Machine Learning Research 2007;8(May):985–1005.
53. Cortes C, Mohri M, Riley M, Rostamizadeh A. Sample selection bias correction theory. In: International conference on algorithmic learning theory Springer; 2008. p. 38–

53.
54. Hernán MA, Hernández-Díaz S, Robins JM. A structural approach to selection bias. Epidemiology 2004;p. 615–625.
55. Austin PC. An introduction to propensity score methods for reducing the effects of confounding in observational studies. Multivariate behavioral research 2011;46(3):399–424.
56. Sugiyama M, Kawanabe M. Machine learning in non-stationary environments: Introduction to covariate shift adaptation. MIT press; 2012.
57. Sun B, Feng J, Saenko K. Return of frustratingly easy domain adaptation. In: Thirtieth AAAI Conference on Artificial Intelligence; 2016. .
58. Huang J, Gretton A, Borgwardt K, Schölkopf B, Smola AJ. Correcting sample selection bias by unlabeled data. In: Advances in neural information processing systems; 2007. p. 601–608.
59. Zhang K, Schölkopf B, Muandet K, Wang Z. Domain adaptation under target and conditional shift. In: International Conference on Machine Learning; 2013. p. 819–827.
60. Sugiyama M, Nakajima S, Kashima H, Buenau PV, Kawanabe M. Direct importance estimation with model selection and its application to covariate shift adaptation. In: Advances in neural information processing systems; 2008. p. 1433–1440.
61. Kanamori T, Hido S, Sugiyama M. A least-squares approach to direct importance estimation. The Journal of Machine Learning Research 2009;10:1391–1445.
62. Courty N, Flamary R, Tuia D, Rakotomamonjy A. Optimal transport for domain adaptation. IEEE transactions on pattern analysis and machine intelligence 2016;39(9):1853–1865.
63. Niculescu-Mizil A, Caruana R. Predicting good probabilities with supervised learning. In: Proceedings of the 22nd international conference on Machine learning; 2005. p. 625–632.

# A Definition and estimation of importance weights

We will implicitly assume that all the random variables we consider admit densities and denote $p_s$ and $p_t$ the density of the joint distribution of $(X, Y)$ applied to the source and target populations respectively. If the support of $p_t$ is included in that of $p_s$ (meaning that $p_s > 0$ wherever $p_t > 0$), we have:

$$\mathbb{E}_{\text{source}}[\,L(Y, f(X))\,] = \mathbb{E}_{\text{target}}\left[\frac{p_t(X,Y)}{p_s(X,Y)}\,L(Y, f(X))\right] , \tag{5}$$

where $L$ is the cost function and $f$ is a prediction function, $\mathbb{E}_{\text{source}}$ (resp. $\mathbb{E}_{\text{target}}$) the expectation on the source (resp. target) data. The risk (on target data) can therefore be computed as an expectation on the source distribution where the loss function is reweighted by the *importance weights*:

$$w(x, y) = \frac{p_t(x, y)}{p_s(x, y)} . \tag{6}$$

If we have empirical estimates $\hat{w}$ of the importance weights $w$, we can compute the reweighted empirical risk:

$$\hat{R}_{\hat{w}}(f) = \sum_{i=1}^{n} \hat{w}(x_i, y_i)\, L(y_i, f(x_i)) . \tag{7}$$

Rather than weighting examples we can also perform importance or rejection sampling [50, 51]. Importances can also be taken into account for model selection – for example in Sugiyama et al. [52] examples of the test set are also reweighted when computing cross-validation scores. Cortes et al. [53] study how errors in the estimation of the weights affect the prediction performance.

## A.1 Preferential Sample selection and Inverse Probability weighting

In the case of preferential sample selection (Section 4), the condition that requires for the support of $p_t$ to be included in the support of $p_s$ translates to a requirement that all individuals have a non-zero probability of being selected: $P(S = 1 \mid x, y) > 0$ for all $(x, y)$ in the support of $p_t$. When this is verified, by applying Bayes' rule the definition of importance weights in Equation (6) can be reformulated [see 53, Sec. 2.3]:

$$w(x, y) = \frac{P(S = 1)}{P(S = 1 \mid X = x, Y = y)} \tag{8}$$

These weights are sometimes called Inverse Probability weights [54] or Inverse Propensity

scores [55]. Training examples that had a low probability of being selected receive higher weights, because they have to account for similar individuals who were not selected.

## A.2 Computing importance weights

In practice we do not know $p_t(x, y)$, which is the joint density of $(X, Y)$ in the target data. However, we do not need it to estimate $p_t/p_s$. More efficient estimation hinges on two observations: we do not need to estimate both densities separately to estimate their ratio, and we can factor out variables that have the same distribution in source and target data.

Here we describe methods that estimate the true importance weights $p_t/p_s$, but we point out that reweighting the training examples reduces the bias of the empirical risk but increases the variance of the estimated model parameters. Even when the importances are perfectly known, it can therefore be beneficial to regularize the weights [43].

***Computing importance weights does not require distributions densities estimation***

Importance weights can be computed by modelling separately $p_s$ and $p_t$ and then computing their ratio [56, Sec. 4.1]. However, distribution density estimation is notoriously difficult; non-parametric methods suffer from the curse of dimensionality and parametric methods depend heavily on the correct specification of a parametric form.

But estimating both densities is more information than we need to compute the sample weights. Instead, we can directly optimize importance weights in order to make the reweighted sample similar to the target distribution, by matching moments [57] or mean embeddings [58, 59], minimizing the KL-divergence [60], solving a least-squares estimation problem [61] or with optimal transport [62].

Alternatively, a discriminative model can be trained to distinguish source and target examples. In the specific case of preferential sample selection, this means estimating directly the probability of selection $P(S = 1)$ (cf Equation (8)). In general, the shift is not always due to selection: the source data is not necessarily obtained by subsampling the target population. In this case we denote $T = 1$ if a subject comes from the target data and $T = 0$ if it comes from the source data. Then, a classifier can be trained to predict from which dataset (source or target) a sample is drawn, and the importance weights obtained from the predicted probabilities [56, Sec. 4.3]:

$$w(x, y) = \frac{P(T = 1 \mid X = x, Y = y)\, P(T = 0)}{P(T = 0 \mid X = x, Y = y)\, P(T = 1)}\,, \quad (9)$$

The classifier must be calibrated (i.e. produce accurate probability estimates, not only a correct decision), see Niculescu-Mizil and Caruana [63]. Note that constant factors such as $P(T = 0)/P(T = 1)$ usually do not matter and are easy to estimate if needed. This discriminative approach is effective because the distribution of $(T \mid X = x, Y = y)$ is much easier to estimate than the distribution of $(X, Y \mid T = t)$ : $T$ is a single binary variable whereas $(X, Y)$ is high-dimensional and often continuous.

The classifier does not need to distinguish source and target examples with high accuracy. In the ideal situation of no dataset shift, the classifier will perform at chance level. On the contrary, a high accuracy means that there is little overlap between the source and target distributions and the biomarker will probably not generalize well.

***What distributions differ in source and target data?***

We may exploit prior information telling us that some distributions are left unchanged in the target data. For example,

$$\frac{p_t(x, y)}{p_s(x, y)} = \frac{p_t(y \mid x)\, p_t(x)}{p_s(y \mid x)\, p_s(x)}\,. \quad (10)$$

Imagine we know that the marginal distribution of input $X$ differs in source and target data, but the conditional distribution of the output $Y$ given the input stays the same: $p_t(x) \neq p_s(x)$ but $p_t(y \mid x) = p_s(y \mid x)$ (a setting known as *covariate shift*). Then, the importance weights simplify to

$$w(x, y) = \frac{p_t(x)}{p_s(x)}\,. \quad (11)$$

In this case, importance weights can be estimated using only unlabelled examples (individuals for whom we do not know $Y$) from the target distribution.

Often, the variables that influence selection (e.g. demographic variables such as age) are lower-dimensional than the full features

(e.g. high-dimensional images), and dataset shift can be corrected with limited information on the target distribution, with importance weights or otherwise. Moreover, even if we have access to additional information $Z$ that predicts selection but is independent of $(X, Y)$, we should *not* use it to compute the importance weights. Indeed, this would only increase the weights' variance without reducing the bias due to the dataset shift [20, Sec. 15.5].

## B Glossary

Here we provide a summary of some terms and notations used in the paper.

**Target population** the population on which the biomarker (machine-learning model) will be applied.

**Source population** the population from which the sample used to train the machine-learning model is drawn.

**Selection** in the case that source data are drawn (with non-uniform probabilities) from the target population, we denote by $S = 1$ the fact that an individual is selected to enter the source data (e.g. to participate in a medical study).

**Provenance of an individual** when we are provided with samples from both the source and the target populations (e.g. Appendix A.2), we also denote $T = 1$ if an individual comes from the target population and $T = 0$ if they come from the source population.

Click here to access/download
**Supplementary Material**
README.txt

Click here to access/download
**Supplementary Material**
sample_selection_bias.py

Click here to access/download
**Supplementary Material**
deconfounding.py

Click here to access/download
**Supplementary Material**
selecting_on_parent_or_child_same_marginals.py

Click here to access/download
**Supplementary Material**
label_shift_2d.py

Click here to access/download
**Supplementary Material**
analysis.py

Click here to access/download
**Supplementary Material**
plotting.py

Click here to access/download
**Supplementary Material**
datasets.py

Click here to access/download
**Supplementary Material**
parabolas_correction_and_shift.py

Click here to access/download
**Supplementary Material**
config.py

Click here to access/download
**Supplementary Material**
selecting_on_parent_or_child.py

Click here to access/download
**Supplementary Material**
label_shift.py

Click here to access/download
**Supplementary Material**
importance_weighting_positivity.py

Click here to access/download
**Supplementary Material**
deconfounding_vary_mi.py

Click here to access/download
**Supplementary Material**
label_shift_naive_bayes.py

Click here to access/download
**Supplementary Material**
deconfounding_3d.py

Click here to access/download
**Supplementary Material**
covariate_shift.py

Click here to access/download
**Supplementary Material**
deconfounding_parabolas.py

Click here to access/download
**Supplementary Material**
sample_selection_bias_continuous_density.py

Click here to access/download
**Supplementary Material**
__init__.py

Click here to access/download
**Supplementary Material**
requirements.txt

cover letter Click here to access/download;Personal Cover;cover.pdf

Dear editors of GigaScience,

we would like to submit a didactic review on dataset shift when defining biomarkers with machine learning, a major threat to external validity of these biomarkers.

Machine-learning techniques are increasingly used to define biomarkers from complex measurements. They hold strong promises for biology and healthcare, such as improving clinical practice and precision medicine with early detection of diseases, or defining intermediate outcomes in epidemiology. However, medical research cohorts often fail to faithfully represent the target population, due to biases such as sample selection biases – the sampling distribution of these datasets is *shifted* with respect to the population that might benefit from the biomarker. This external-validity challenge is seldom discussed in the context of machine-learning practice. Yet, such settings can break standard machine-learning tools: the extracted biomarker may not perform well on the target population.

We think that a didactic review on this topic is important and timely given the increasing number of publications that opportunistically apply machine-learning techniques to biomedical datasets. While machine-learning methods carry great promises for medicine and public health, they are often developed without properly taking dataset shift into account, applied without measuring how much this shift limits their validity, or discarded without resorting to appropriate techniques to make them more robust. In addition, the literature contains some misunderstanding regarding the solutions to dataset shift, as intuitions do not carry over from inferential statistics to predictive modeling. The specific focus of our proposed review is to explain progress in mathematical techniques to non specialists who can most benefit from them, namely healthcare researchers.

Best regards,

Jérôme Dockès, Gaël Varoquaux, Jean-Baptiste Poline.
