# Supplementary material for: Preventing dataset shift from breaking machine-learning biomarkers
Source: Gigascience. 2021 Sep 28;10(9):giab055. doi: 10.1093/gigascience/giab055 (PMC8478611; doi:10.1093/gigascience/giab055)
Supplement: giab055_GIGA-D-21-00081_Revision_1 [file giab055_giga-d-21-00081_revision_1.pdf]

GigaScience

Preventing dataset shift from breaking machine-learning biomarkers

--Manuscript Draft--

**Manuscript Number:**	GIGA-D-21-00081R1
**Full Title:**	Preventing dataset shift from breaking machine-learning biomarkers
**Article Type:**	Review
**Funding Information:**	National Institutes of Health (NIH-NIBIB P41 EB019936) — Dr Jean-Baptiste Poline<br>National Institute of Mental Health (NIH-NIMH R01 MH083320) — Dr Jean-Baptiste Poline<br>National Institutes of Health (NIH RF1 MH120021) — Dr Jean-Baptiste Poline<br>National Institute of Mental Health (R01MH096906) — Dr Jean-Baptiste Poline
**Abstract:**	Machine learning brings the hope of finding new biomarkers extracted from cohorts with rich biomedical measurements. A good biomarker is one that gives reliable detection of the corresponding condition. However, biomarkers are often extracted from a cohort that differs from the target population. Such a mismatch, known as a dataset shift, can undermine the application of the biomarker to new individuals. Dataset shifts are frequent in biomedical research, e.g. because of recruitment biases. When a dataset shift occurs, standard machine-learning techniques do not suffice to extract and validate biomarkers. This article provides an overview of when and how dataset shifts breaks machine-learning extracted biomarkers, as well as detection and correction strategies.
**Corresponding Author:**	Jérôme Dockès<br>McGill University<br>Montréal, CANADA
**Corresponding Author Secondary Information:**	
**Corresponding Author's Institution:**	McGill University
**Corresponding Author's Secondary Institution:**	
**First Author:**	Jérôme Dockès
**First Author Secondary Information:**	
**Order of Authors:**	Jérôme Dockès<br>Gaël Varoquaux<br>Jean-Baptiste Poline
**Order of Authors Secondary Information:**	
**Response to Reviewers:**	To make it easier to read, we have prepared our response to reviewer and editor comments as a pdf file, attached to the new submission -- reply.pdf
**Additional Information:**	
**Question**	**Response**
Are you submitting this manuscript to a special series or article collection?	No
**Experimental design and statistics**<br><br>Full details of the experimental design and	Yes

Powered by Editorial Manager® and ProduXion Manager® from Aries Systems Corporation

statistical methods used should be given in the Methods section, as detailed in our Minimum Standards Reporting Checklist. Information essential to interpreting the data presented should be made available in the figure legends.<br><br>Have you included all the information requested in your manuscript?	
**Resources**<br><br>A description of all resources used, including antibodies, cell lines, animals and software tools, with enough information to allow them to be uniquely identified, should be included in the Methods section. Authors are strongly encouraged to cite Research Resource Identifiers (RRIDs) for antibodies, model organisms and tools, where possible.<br><br>Have you included the information requested as detailed in our Minimum Standards Reporting Checklist?	Yes
**Availability of data and materials**<br><br>All datasets and code on which the conclusions of the paper rely must be either included in your submission or deposited in publicly available repositories (where available and ethically appropriate), referencing such data using a unique identifier in the references and in the "Availability of Data and Materials" section of your manuscript.<br><br>Have you have met the above requirement as detailed in our Minimum Standards Reporting Checklist?	Yes

Powered by Editorial Manager® and ProduXion Manager® from Aries Systems Corporation

Response to reviewer and editor comments

We thank the editor and the reviewers for their thoughtful comments. We describe below how we have addressed them in the revised manuscript

1 Editor

Real data *In particular, all reviewers feel that an example with real data should be included.*

Reply We thank the editor and the reviewers for suggesting an example on real data. We added such an example, drawing samples with different age distributions from the UKBiobank dataset, and studying the task of predicting smoking status.

By choosing a large dataset and a classification task with well balanced classes (as opposed to disease diagnostics where one class is usually much smaller), we ensure that we have plenty of data. This enables us to subsample it to draw samples with different age distributions, then further divide it into cross-validation splits, and still obtain test sets that are large enough for the prediction scores to be meaningful.

The results of this experiment illustrate several of the points we discuss in the review and show with simulated data (please see details in Section 3, Figure 2 and Appendix B):

- in this example the dataset shift does degrade prediction performance
- regressing out age (the variable whose distribution changes) does not help to handle the dataset shift and is detrimental in all configurations.
- the dataset shift affects the linear model as much as the non-linear model: strong constraints are not a solution to dataset shift and with sufficient sample size the powerful learner performs much better with or without dataset shift.
- Importance Weighting seems to improve the scores of the linear model in the presence of dataset shift.
- However Importance Weighting degrades the performance of the best model (the gradient boosting) in the presence of dataset shift. Indeed, the non-linear model can already learn flexible boundaries and rely on local information to classify individuals of a certain age group. Downweighting participants from the over-represented age group increases the risk of overfitting without bringing important benefits, so Importance Weighting degrades performance. Note that we observe a similar behaviour in the simulated data in Figure 1: IW improves the boundary for the target population only for the linear model.
- Therefore for this example the best approach is to ensure the whole support of the target distribution is represented in the training data (even if the distribution is shifted), and rely on a large dataset and powerful machine-learning model.

public repository *On an editorial note, your python files are included as supplemental files at the moment, I recommend to share them via a code repository instead and cite the repo in the paper. Please also add license info for the code (OSI-approved licences here: `https://opensource.org/licenses`).*

Reply Thank you for this suggestion. We have created a repository containing all the sources for this paper (and added an MIT license): `https://github.com/neurodatascience/dataset_shift_biomarkers`. We have added a paragraph indicating this, “Software and data availability”, at the end of the conclusion.

2 Reviewer 1

summary *This paper addresses a very important and often undermined challenge in deriving new biomarkers for disease using machine learning techniques. Very often, new methods are limited to validation experiments with cross-validation or using training and testing datasets with similar characteristics. Also, datasets used in validations are often affected by selection bias. Thus, these experiments may not provide a realistic evaluation of application to new individuals, e.g. in a clinical setting. The paper describes possible biases in data used for training and testing, describes the effects of "dataset shift" on the accuracy of final biomarkers, and presents techniques to deal with dataset shift.*

The paper is well written in general. I enjoyed reading it as a tutorial that briefly presents the basic concepts and then incrementally introduces the main problem. The illustration of the dataset shift problem using toy examples and visualizations is very useful. After a very clear introduction and problem description, the paper presents a generic tool to address this problem. The proposed solution, importance weighting, is not novel. However, presenting it in this context was informative. Section 6.1 (covariate shift) nicely links to importance weighting technique. However, I found that section 6.2 was disconnected, so maybe it would require a more clear description and a careful discussion.

section 6.2 disconnected *However, I found that section 6.2 was disconnected, so maybe it would require a more clear description and a careful discussion.*

Reply Thank you for pointing this out. To include it better to the rest of the paper, we now point to this section (is now 7.2, "prior probability shift") from the introduction of section 7, and we have expanded it to include more discussion of class imbalance and highlight that this special case of dataset shift is easy to correct, which is why we deemed it deserved a special mention.

2.1 A few major comments:

example with real data *The paper reads well as a concept paper; however, it does not include any examples with real data. Toy examples for illustrations are very informative. However, examples on real datasets with quantitative evaluations would be necessary to show the effects of dataset shift in real problems, and to show how the proposed approach actually works. I think that this is the major missing part in the paper. Addition of results using real datasets would significantly increase the value of the paper, particularly if they can be selected in a way that will illustrate the problems mentioned in the paper.*

Reply Thank you for suggesting such an experiment. We have included an example relying on the UKBiobank data (predicting the smoking status of participants, and using training and testing sets with different age distributions). Please see the second paragraph of Section 3, Figure 2, Appendix B, and our reply to the editor.

data heterogeneity *A major challenge in deriving imaging biomarkers is to handle heterogeneity of imaging data and clinical labels due to various factors, such as scan parameters/protocols and variability of measuring protocols used. The paper did not discuss how to handle data heterogeneity, which is another major source for dataset shift. Only in section 3 there is a suggestion to use heterogeneous sets for training (which I agree), but it's not clear how to derive robust biomarkers in the presence of heterogeneous datasets (e.g. there is no mention of data harmonization). I think this is a limitation for the paper.*

Reply We agree that changes due to sites, imaging devices, acquisition parameters etc. constitute a major challenge. We now provide more discussion in the beginning of section 6 and a new dedicated section, 6.2, "multi-site datasets". We mention possible approaches, such as minimizing the loss on the worst site or scanner to encourage more robust estimation, or the learning of invariant features. However we

believe that learning robust biomarkers despite the heterogeneity of parameters and measuring protocols remains an open problem.

2.2 One minor comment that may help to improve the paper:

In section 3, after reading the first sentence, I had the impression that the listed items in italic were the "misconceptions", so they are showing what is wrong/incorrect. I had to go back and read them again after I noticed that it was the opposite. I would suggest the authors to edit this part in a way that will remove the ambiguity.

Reply Thank you for pointing out this confusing wording. We have reworked the section title and its wording to remove this ambiguity.

3 Reviewer 2

summary *This article covers a vitally important topic in machine learning generally and specifically its application to healthcare and life science. The mismatching of attributes and properties in training and testing data is a significant issue. The authors raise these important issues and present some ideas and methods for how to address what they call 'dataset drift'.*

emphasize fig 3 *Figure 3 and the corresponding text are of interest and this should be emphasized more than it is currently.*

Reply We agree that the concrete consequences of the causal relations underlying the data on machine learning models' generalization is an important and interesting topic. However it is a rather technical and difficult one, and we wanted to keep the paper very accessible, which is why we chose to simply introduce the issue very briefly and provide references for interested readers. To emphasize this topic slightly more, we have added another reference to a publication aiming to exploit causal knowledge to improve domain adaptation, robustness to dataset shift at the end of Section 4.1.

3.1 major comments

There are several areas where the paper can be improved and given the importance of the topic and target audience, I would strongly recommend the authors consider these changes.

real data *The authors present some examples of dataset drift and possible issues that arise, some from the literature and some from toy examples. I think this would have much stronger impact if a real dataset were used in the paper to demonstrate this.*

Reply Thank you for suggesting such an experiment. We have included an example relying on the UKBiobank data (predicting the smoking status of participants, and using training and testing sets with different age distributions). Please see the second paragraph of Section 3, Figure 2, Appendix B, and our reply to the editor.

class imbalance *The authors refer to 'probability shift' to refer to the difference in populations sizes in the training and testing data, commonly referred to as class-imbalance. This is quite brief in the paper and constitutes one of the biggest issues in machine learning in life sciences and more emphasis should be devoted to this. Explicit refer to class-imbalance (and some references) is required here as there is a large area of research devoted to this. Moreover, the authors must be clear what are the disadvantages of training*

models on balanced data when the population is imbalanced, and how using training data that reflects the population (e.g. with impedance) and then using the proposed methods provide an advantage. Figure 6 could be extended to show this for example. The machine learning community often use balanced training data to avoid 'short cuts' to high accuracies and identify the features need to predict a label given the input. If you have an appropriate model that identifies robust features in training, then the low frequency of a class in the testing / real world data (e.g. rare disease) should not degreed performance. This may also apply to other characteristics / properties in the training data.

Reply We agree that class imbalance is an active area of research in machine learning, and that it is particularly relevant to the life sciences, where having a very strong imbalance is common (for example when learning to diagnose a disease). We now explicitly use the term "class-imbalance" in Section 7.2 and provide a reference to a review on the topic. We also agree that when the classes are well separated, the shift in prior probabilities does not have a strong impact on the posterior – a good classifier will generalize well despite the change in class balance. We also added a sentence regarding this fact.

However, class imbalance in general is a problem of its own, and much of the literature on this topic is not directly related to dataset shift. We therefore prefer to limit our discussion of class imbalance in the case where it does result in a dataset shift – when the prior probability is voluntarily shifted to make classes more balanced in the training data. By avoiding a more complete discussion of class imbalance, we hope to keep our didactic review short and focused on dataset shift.

Transforming input data *Similarity, the authors have not discussed the notion of transforming the data distributions prior to application of a model, e.g. optimal transport. What are the benefits of the proposed methods over these?*

Reply Indeed our discussion was too heavily focused on sample weighting, skimming over other approaches. We now discuss other possibilities in Section 6, including transforming features (possibly relying on optimal transport), learning features that do not discriminate source and target domains with adversarial methods, and data augmentation.

homogeneous training data *The authors state on Line 166 Training examples should not be selected to be homogeneous. This maybe conflating issues from each of the healthcare and ML domains and may not be a general recommendation for all problems, the authors should expand this discussion to justify this recommendation.*

Reply Thank you for pointing out this imprecision. Indeed this was not meant to be a general recommendation for all problems, as in this paper we discuss strictly the predictive setting – and not, for example, statistical testing nor causal inference. To make this clearer, we have added the following sentence to this paragraph:

> Therefore in predictive settings, when we want to ensure a good generalization of machine-learning models, large and diverse datasets are desirable.

Moreover, the paragraph already contained this phrase:

> While this may help reduce variance and improve statistical testing

which we hope indicates that there may be motivations to carefully select homogeneous participants on some settings – but not in the case where the goal is accurate out-of-sample prediction.

biomarker terminology *The authors often refer to the f(x) as the biomarker, this is not correct. F(x) is the model and the biomarkers are the inputs x. The model finds a combination of these to differentiate the classes through f(x). This leads to data (i.e. biomarker) vs model considerations which are not discussed. That is, models will be sensitive to 'dataset shifts', but if biomarkers are robust then they should be invariant to such shifts (at least in theory) with a 'good' model. Additionally, you do not 'build biomarkers', the model identifies them in the data.*

Reply Thank you for pointing out this inaccuracy in terminology. We have now edited the text to refer to "machine-learning models" and "biomarkers identified through machine-learning" rather than referring to a machine-learning model as a biomarker. We now use "identifying a biomarker" rather than "building a biomarker"

IW in main article *Section 5 why has the precise definition and overview in the appendix? This section required more detail as it is currently conceptual only. Further details can be in the appendix, but more are required here.*

Reply We chose to keep the precise description of importance weighting to keep the main text at a conceptual level and focussed on a description of the dataset shift problem rather than the details of a particular solution.

From the reviews we perceived that the paper focussed too heavily on importance weighting, thus we chose to keep the details of importance weighting in the appendix, and add a richer discussion of other solutions in the main text.

3.2 Some minor comments to the authors

notation *Section 2.1 you use lower case x and y but have not defined them (as individual instances in X and Y). one small sentence will suffice, you do this in line 89 but this should be earlier. You also use X and Y for the seen and unseen data on line 83.*

Reply Thank you for pointing out this omission. To keep the discussion conceptual and avoid introducing extra notation, we have now decided to use X and Y only, which are defined at the beginning of Section 2.1.

wording *The first entice in section 2.2 line 111 does not read well and the citation doesn't relate to a statement clearly. Training performance only is not just an 'optimistic' estimate it is potentially meaningless due to what you are calling data shit and the fact that some ML methods (eg neural nets) can fit any arbitrary data and hence overfit.*

Reply We have now reworded that sentence (beginning of section 2.2) and added more details to explain in what overfitting consists. Moreover, we have specified more precisely to which section of the Poldrack et al. paper we refer. It does describe the phenomenon of overfitting:

> For this reason, a model will usually fit better to the sample used to estimate it than it will to a new sample, a phenomenon known in machine learning as overfitting

The Section 7.4 of "the elements of statistical learning", titled "Optimism of the training error rate" also discusses exactly that topic. We consider that these references are complementary because the Poldrack et al provides an intuition of the problem without any mathematical formalism, while the Hastie et al provides a complete explanation.

fig 1 *Fig 1 caption. Age is indicated by shade not colour. Healthy and disease are indicated by colour. Also I assume blue corresponds 'unhealthy' patients as this is not stated and needs to be. It seems that the RBF-SVM could be improved for the source data (for younger patients)*

Reply Thank you for pointing out these imprecisions. The caption now makes it clear that blue circles correspond to unhealthy subjects and we have replaced the erroneous "color" with "shade of gray".

Regarding the rbf SVM performance, our goal was not to find the best possible performance for this toy data but to qualitatively illustrate the different generalization properties of a linear and non-linear model. Therefore we may not have selected the best hyperparameter for this data. In fact, we have selected a rather strong regularization in order to have a simple boundary and more readable figure. As you can see on the figure obtained with a smaller regularization term (below), the learned boundary improves but the conclusions drawn from the figure as a whole do not change.

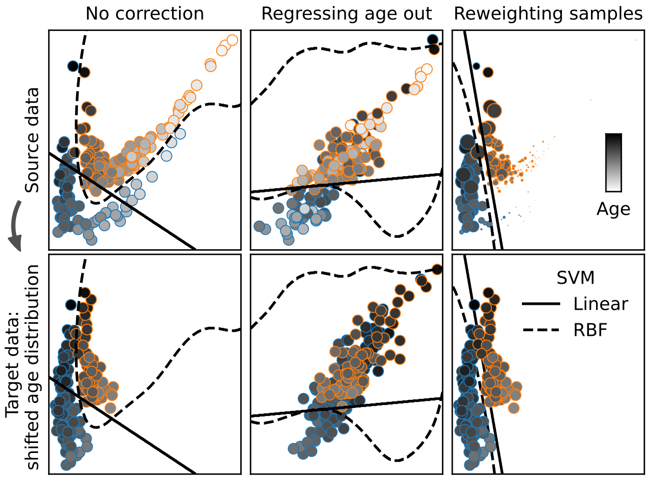

fig 2 *Fig 2 the caption needs more information. what is the shade of the arrows representing? A gradient between younger and older? What to the arrows (and their width represent?) what is the joining arrow indicating?*

Reply Indeed the caption of Figure 2 was too terse. We have added paragraphs to describe the meaning of the arrows, their shade and width, and the small arrow that represents a jump from a Healthy to a Diseased trajectory. As the reviewer assumed, the shade of the arrows represents a gradient from younger to older (as it also does in Figure 1). We have also added color, and merged figures 1 and 2.

figure order *The text should refer to figures in order, currently it refers to figures 1,5,3 . . . and figure 2 is first referred in figure 1's caption.*

Reply Thank you for noting this awkard order. The figures are now referred to in the correct order.

mention of reviews on transfer learning *line 288-289 are not relevant to the rest of the paragraph. You have not mentioned anything to do with transfer learning.*

Reply We agree that the connection of these references with the rest of the paragraph was not clear enough. The reviews on transfer learning are now mentioned in Section 6, "other approaches to dataset shift". We consider that they are relevant because they describe some of the solutions we mention in more detail, as well as other methods. Dataset shift is a subset of transfer learning, and is therefore discussed

in these reviews, which are relevant for readers who want a deeper and more formal discussion of the concepts exposed in our paper.

Mentioning the term "transfer learning" also provides another useful keyword that readers can use to search more information on these topics.

fig 4 *Figure 4 is unnecessary as this is described in the text clearly.*

Reply We agree that Figure 4 is not absolutely essential. However, we feel that the point that all segments of the population must be represented in the training data is crucial, and that emphasizing it with a small illustration may be beneficial, especially to readers who are reading the paper quickly. Moreover, this figure takes very little space and we hope it can be understood quickly.

4 Reviewer 3:

summary *This manuscript presents a didactic review which discusses the implications of dataset shift when designing and evaluating machine learning-based biomarkers. The review covers some basic machine learning concepts (empirical risk minimization, evaluation practices, etc), points out common misconceptions, characterizes different types of dataset shift, and discusses importance weighting as a potential solution to this problem.*

The paper is nicely written, didactic and clear. I feel it is also timely since currently many researchers who are not coming from the fields of statistics or machine learning are using such methods to analyze their own datasets and derive biomarkers for various pathologies. Overall, the manuscript is interesting but I think it would benefit from including a few more concrete examples, linking the theoretical concepts to applications in the context of biomarkers (see some of my comments below).

4.1 major comments

I have some recommendations that may help to improve the quality of the paper:

scanner differences *One common source of dataset shift in the context of image-derived biomarkers is related to the equipment brand or configuration parameters used to capture such images (e.g. the MR or CT machine used to perform the studies). I would like the authors to discuss this fact and link it to the concepts introduced in the review.*

Reply Indeed differences between scanners can be an important challenge. We have added Section 6.2 which mentions potential approaches such as learning on multi-site or multi-scanner datasets while minimizing the loss on the worst site and also mention learning invariant representations (beginning of Section 6).

additional concepts to define *To me, the target audience for this didactic review is mostly healthcare and biomedical researchers, who are using machine learning and data analysis tools to produce novel biomarkers. In that sense, I would not take for granted the fact that the audience is familiar with basic concepts like 'confounding' for example, which are used but not defined. Another important term which is used but not discussed is 'fairness' (line 176). Since this is an educational review, I recommend the authors to devote a few lines to introduce such concepts and discuss them in the context of predictive models.*

Reply Indeed this comment is very relevant as our review is mostly intended for healthcare and biomedical researchers. We now provide a brief definition of confounding at the beginning of section 3 and in the Glossary (Appendix C). Fairness in the general context of machine learning has several tentative definitions and we prefer not to dive into details to avoid diluting the focus of the paper, but we did try to clarify what we mean by it in the context of biomarkers' prediction performance in the second paragraph of Section 3.

fig 2 *The thick arrows in Figure 2 are a bit confusing. What does the direction of the thick arrows indicate?. Also, g and h are not specified. The Figure just indicates "for some g,h". What do g and h represent? What is their actual form?*

Reply The direction of the thick arrows represents increasing age – the arrows schematize the trajectory of ageing subjects across the 2-dimensional feature plane. Their width represents the size of each group (Healthy and Diseased): the Healthy group is large at young ages, and the Healthy group diminishes and the Diseased group grows as age increases. We have reworked the figures and expanded the caption to better describe the meaning of the arrows, their width, and the grayscale gradient. (Note figures 1 and 2 have been merged).

The goal of the equations with g and h was only to explain that Y depends only on age, and X depends on both Y and age. As the actual form of these dependencies is not important and we realized these equations added ore confusion than clarity, we removed them.

concrete scenarios for IW *The authors focus on importance weighting as a tool to deal with dataset shift, and dedicate a complete appendix to its definition. However, as discussed in section 7, importance weighting needs a clear definition of the targeted population and access to a diverse dataset, which may not be the case in real scenarios. Since this review is related to biomarkers, I think it would be important to discuss a few more concrete and real scenarios (beyond the examples shown in the figures) where importance weighting could be used to mitigate dataset shift in the context of machine-learning based biomarkers.*

Reply We agree that importance weighting may not be applicable, or may not improve prediction, in a variety of scenarios. We have expanded the section on importance weighting and the conclusion to insist more on the limitations of importance weighting (see the last paragraph of Section 5 in particular).

However, importance weighting can be helpful in the case of a covariate shift when the learner underfits – when the model is misspecified, eg when a linear model is used to approximate a non-linear function. We think that this situation does happen in practice, as in the left panel of the new Figure 2, or the cited examples on UCI datasets for breast cancer and heart disease prediction (although we realize these are illustrations on small datasets rather than genuine biomedical studies).

Another reason why we chose to give special treatment to importance weighting is that, unlike many other approaches, it is conceptually simple, easy to implement, and usually does not incur an important computational cost. It is therefore particularly relevant to healthcare and biomedical researchers who are interested in solutions that can readily be deployed on datasets of a realistic size. Our recommendation is to include an importance-weighted estimator in the set of candidates to be tested (and compared to a baseline without any dataset shift adaptation) on real data from the target distribution, when feasible. We state the recommendation of using a non-weighted baseline in the second paragraph of Section 6.

complete section for other solutions *As previously discussed, importance weighting is somehow limited in situations where we do not have a clear definition of the targeted population. In fact, the authors discuss in the Conclusions section alternative approaches that may be used when this is not possible (e.g. distributionally robust optimization). Why not devoting a complete section to discuss alternative approaches*

useful in the absence of information about the target population? I am not sure if the Conclusions section is the right place to introduce them.

Reply We have now added Section 6, "other approaches to dataset shift". It does not provide technical details but gives an overview of other solutions, including invariant features and adversarial domain adaptation, data augmentation, robust optimization, and loss variance reduction. It also discusses the case of datasets collected across several sites or imaging devices.

experiments with real data *As far as I understand, all the examples provided in the manuscript are coming from synthetic data. I think for a didactic review like this one, and specially for a journal like Gigascience, it would be nice to provide a case study using real data, which reflects a dataset shift between source and target, that can be corrected using the discussed importance weighting strategy. It doesn't have to be a huge dataset, just a simple case with concrete features X and labels Y. Maybe using some public database of tabulated samples which illustrates a real scenario?*

Reply Thank you for suggesting such an experiment. We have included an example relying on the UKBiobank data (predicting the smoking status of participants, and using training and testing sets with different age distributions). Please see the second paragraph of Section 3, Figure 2, Appendix B, and our reply to the editor.

note *General Note: I think it is important to acknowledge the fact that I am coming from the computer vision and medical image computing community, not from statistics or causal analysis, and thus my comments in that regard may be limited.*

4.2 Minor comment:

typo *Line 385: "Importance weighting needs a clear definition the targeted population". It should be "Importance weighting needs a clear definition of the targeted population".*

Reply Thank you for pointing this out; we have corrected that typo.

Revised manuscript Click here to access/download;Manuscript;main.pdf

OXFORD (GIGA)n SCIENCE

GigaScience, 20xx, 1–10

doi: xx.xxxx/xxxx
Manuscript in Preparation
Paper

PAPER

Preventing dataset shift from breaking machine-learning biomarkers

Jérôme Dockès[1, *], Gaël Varoquaux[1, 2,†] and Jean-Baptiste Poline[1, †]

[1]McGill University and [2]INRIA

[*]Corresponding author.
[†]JB Poline and Gaël Varoquaux contributed equally to this work.

Abstract

Machine learning brings the hope of finding new biomarkers extracted from cohorts with rich biomedical measurements. A good biomarker is one that gives reliable detection of the corresponding condition. However, biomarkers are often extracted from a cohort that differs from the target population. Such a mismatch, known as a dataset shift, can undermine the application of the biomarker to new individuals. Dataset shifts are frequent in biomedical research, e.g. because of recruitment biases. When a dataset shift occurs, standard machine-learning techniques do not suffice to extract and validate biomarkers. This article provides an overview of when and how dataset shifts breaks machine-learning extracted biomarkers, as well as detection and correction strategies.

1 Introduction: dataset shift breaks learned biomarkers

Biomarkers are measurements that provide information about a medical condition or physiological state [1]. For example, the presence of an antibody may indicate an infection; a complex combination of features extracted from a medical image can help assess the evolution of a tumor. Biomarkers are important for diagnosis, prognosis, and treatment or risk assessments.

Complex biomedical measures may carry precious medical information, as with histopathological images or genome sequencing of biopsy samples in oncology. Identifying quantitative biomarkers from these requires sophisticated statistical analysis. With large datasets becoming accessible, supervised machine learning provides new promises by optimizing the information extracted to relate to a specific output variable of interest, such as a cancer diagnosis [2, 3, 4]. These methods, cornerstones of artificial intelligence, are starting to appear in clinical practice: a machine-learning based radiological tool for breast-cancer diagnosis has recently been approved by the FDA[1].

Can such predictive biomarkers, extracted through complex data processing, be safely used in clinical practice, beyond the initial research settings? One risk is the potential mismatch, or *dataset shift*, between the distribution of the individuals used to estimate this statistical link and that of the target population that should benefit from the biomarker. In this case, the extracted associations may not apply to the target population [5]. Computer aided diagnostic of thoracic diseases from X-ray images has indeed been shown to be unreliable for individuals of a given sex if built from a cohort over-representing the other sex [6]. More generally, machine-learning systems may fail on data from different imaging devices, hospitals, populations with a different age distribution, *etc.*. Dataset biases are in fact frequent in medicine. For instance selection biases –*eg* due to volunteering self-selection, non-response, dropout...– [7, 8] may cause cohorts to capture only a small range of possible patients and disease manifestations in the presence of spectrum effects [9, 10]. Dataset shift or dataset bias can cause systematic errors that cannot be fixed by acquiring larger datasets and require specific methodological care.

In this article, we consider predictive biomarkers identified with supervised machine learning. We characterize the problem of dataset shift, show how it can hinder the use of machine learning for health applications [11, 12], and provide mitigation strategies.

1 https://fda.report/PMN/K192854

Compiled on: July 6, 2021.
Draft manuscript prepared by the author.

2 A primer on machine learning for biomarkers

2.1 Empirical Risk Minimization

Let us first introduce the principles of machine learning used to identify biomarkers. Supervised learning captures, from observed data, the link between a set of input measures (features) X and an output (e.g. a condition) Y: for example the relation between the absorption spectrum of oral mucosa and blood glucose concentration [13]. A supervised learning algorithm finds a function f such that $f(X)$ is as close as possible to the output Y. Following machine-learning terminology, we call the system's best guess $f(X)$ for a value X a *prediction*, even when it does not concern a measurement in the future.

Empirical Risk Minimization, central to machine learning, uses a loss function L to measure how far a prediction $f(X)$ is from the true value Y, for example the squared difference:

$$L(Y, f(X)) = (Y - f(X))^2 \ . \tag{1}$$

The goal is to find a function f that has a small *risk*, which is the *expected* loss on the true distribution of X and Y, i.e. on *unseen individuals*. The true risk cannot be computed in practice: it would require having seen all possible patients, the true distribution of patients. The *empirical* risk is used instead: the average error over available examples,

$$\hat{R}(f) = \frac{1}{n} \sum_{i=1}^{n} L(y_i, f(x_i)) \ , \tag{2}$$

where $\{(x_i, y_i) \ , \ i = 1, \ldots, n\}$ are available (X, Y) data, called *training* examples. The statistical link of interest is then approximated by choosing f within a family of candidate functions as the one that minimizes the empirical risk $\hat{R}(f)$.

The crucial assumption underlying this very popular approach is that the prediction function f will then be applied to individuals drawn from the same population as the training examples $\{x_i, y_i\}$. It can be important to distinguish the *source* data, used to fit and evaluate a machine-learning model (e.g. a dataset collected for research), from the *target* data, on which predictions are meant to be used for clinical applications (e.g. new visitors of a hospital). Indeed, if the training examples are not representative of the target population – if there is a dataset shift – the empirical risk is a poor estimate of the expected error, and f will not perform well on individuals from the target population.

2.2 Evaluation: Independent test set and cross-validation

Once a model has been estimated from training examples, measuring its error on these same individuals results in a (sometimes wildly) optimistic estimate of the expected error on *unseen* individuals (Friedman et al. [14, Sec. 7.4], Poldrack et al. [15, Sec. 1, "Association vs Prediction"]). Indeed, predictors chosen from a rich family of functions are very flexible and can learn rules that fit tightly the training examples but fail to generalize to new individuals. This is called *overfitting*.

To obtain valid estimates of the expected performance on new data, the error is measured on an independent sample held out during training, called the test set. The most common approach to obtain such a test set is to randomly split the available data. This process is usually repeated with several splits, a procedure called cross-validation [16, 14, Sec. 7].

When training and test examples are chosen uniformly from the same sample, they are drawn from the same distribution (i.e. the same population): there is no dataset shift. Some studies also measure the error on an *independent* dataset [e.g. 17, 18]. This helps establishing external validity, assessing whether the predictor will perform well outside of the dataset used to define it [19]. Unfortunately, the biases in participant recruitment may be similar in independently collected datasets. For example if patients with severe symptoms are difficult to recruit, this is likely to distort all datasets similarly. Testing on a dataset collected independently is therefore a useful check, but no silver bullet to rule out dataset shift issues.

3 False solutions to tackling dataset shift

We now discuss some misconceptions and confusions with problems not directly related to dataset shift.

"Deconfounding" does not correct dataset shift for predictive models. Dataset shift is sometimes confused with the notion of *confounding*, as both settings arise from an undesired effect in the data. Confounding comes from *causal analysis*, estimating the effect of a *treatment* –an intervention, sometimes fictional– on an outcome. A confounder is a third variable –for example age, or a comorbidity– that influences both the treatment and the outcome. It can produce a non-causal association between the two [See 21, Chap. 7, for a precise definition]. However, the machine-learning methods we consider here capture statistical associations, but *do not target causal effects*. Indeed, for biomarkers, the association itself is interesting, whether causal or not. Elevated body temperature may be the consequence of a condition, but also cause a disorder. It is a clinically useful measure in both settings.

Tools for causal analysis are not all useful for prediction, as pointed out by seminal textbooks: "if the goal of the data analysis is purely predictive, no adjustment for confounding is necessary [...] the concept of confounding does not even apply."[21, Sec. 18.1], or Pearl [22]. In prediction settings, applying procedures meant to adjust for confounding generally degrades prediction performance without solving the dataset shift issue. Figure 1 demonstrates the detrimental effect of "deconfounding" on simulated data: while the target population is shifted due to a different age distribution, removing the effect of age also removes the separation between the two outcomes of interest. The same behavior is visible on real epidemiologic data with age shifts, such as predicting the smoking status of participants in the UKBiobank study [23], as shown in Figure 2. Drawing training and testing samples with different age distributions highlights the effect of these age shifts on prediction performance (see Appendix B for details on the procedure). For a given learner and test population, training on a different population degrades prediction. For example, predictions on the old population are degraded when the model is trained on the young population. A flexible model (Gradient Boosting) outperforms the linear model with or without dataset shift. "Regressing out" the age (as in the second column of Figure 1, "+ regress-out" strategy in Figure 2) degrades the predictions in *all* configurations.

For both illustrations on simulated and real data (Figure 1 and 2), we also demonstrate an approach suitable for predictive models: reweighting training examples giving more importance to those more likely in the test population. This approach improves the predictions of the overconstrained (misspecified) linear model in the presence of dataset shift, but degrades the predictions of the powerful learner. The non-linear model already captures the correct separation for both young and old individuals, thus reweighting examples does not bring any benefit but only increases the variance of the empirical risk. A more detailed discussion of this approach, called *importance weighting*, is provided in Section 5.

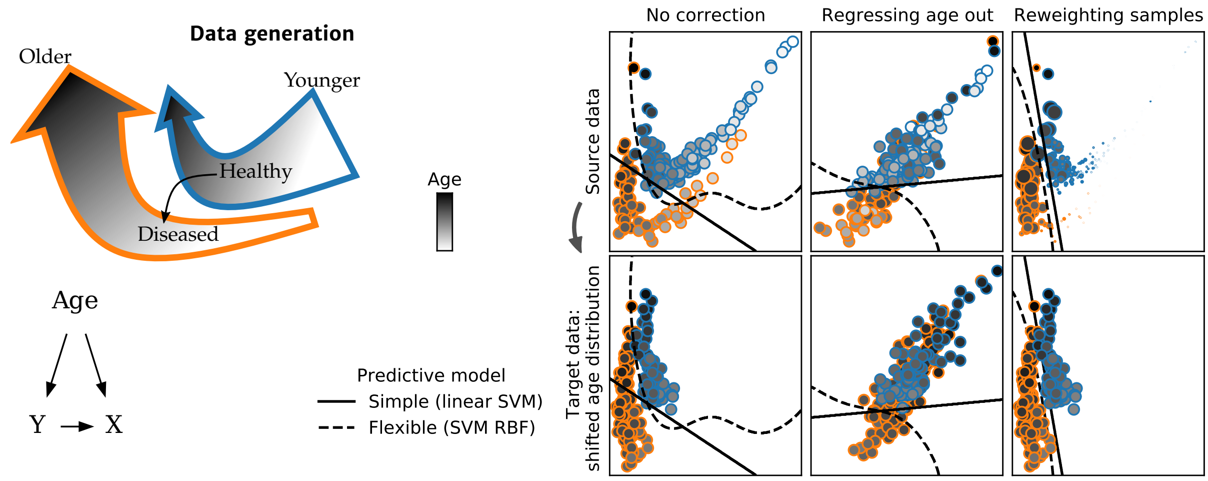

Figure 1. Classification with dataset shift – regressing out a correlate of the shift does not help generalization. The task is to classify patients (orange) from healthy controls (blue), using 2-dimensional features. Age, indicated by the shade of gray, influences both the features and the probability of disease. **Left: generative process for the simulated data.** Age influences both the target Y and the features X, and Y also has an effect on X. Between the source and target datasets, the distribution of age changes. The two arrows point towards increasing age and represent the Healthy and Diseased populations, corresponding to the orange and blue clouds of points in the right panel. The grayscale gradient in the arrows represents the increasing age of the individuals (older individuals correspond to a darker shade). Throughout their life, individuals can jump from the Healthy trajectory to the Diseased trajectory, which is slightly offset in this 2-dimensional feature space. As age increases, the prevalence of the disease increases, hence the Healthy trajectory contains more individuals of young ages (its wide end), and less at older ages (its narrow end) – and vice-versa for the Diseased trajectory. **Right: predictive models** In the target data (bottom row), the age distribution is shifted: individuals tend to be older. Elderly are indeed often less likely to participate in clinical studies [20]. **First column:** no correction is applied. As the situation is close to a covariate shift (Section 7.1), a powerful learner (RBF-SVM) generalizes well to the target data. An over-constrained model – Linear-SVM – generalizes poorly. **Second column:** wrong approach. To remove associations with age, features are replaced by the residuals after regressing them on age. This destroys the signal and results in poor performance for both models and datasets. **Third column:** Samples are weighted to give more importance to those more likely in the target distribution. Small circles indicate younger individuals, with less influence on the classifier estimation. This reweighting improves prediction for the linear model on the older population.

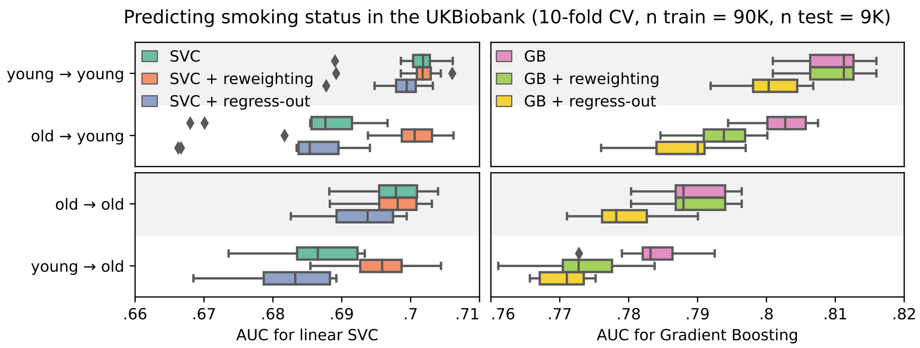

Figure 2. Predicting the smoking status of UKBiobank participants. Different predictive models are trained on 90K UKBiobank participants and tested on 9K participants with a possibly shifted age distribution. "young → old" means the training set was drawn from a younger sample than the testing set. Models perform better when trained on a sample drawn from the same population as the testing set. Reweighting examples that are more likely in the test distribution ("+ reweighting" strategy, known as Importance Weighting, Section 5) alleviates the issue for the simple linear model, but is detrimental for the Gradient Boosting. Regressing out the age ("+ regress-out" strategy) is a bad idea and degrades prediction performance in all configurations.

Training examples should not be selected to be homogeneous. To obtain valid predictive models that perform well beyond the training sample, it is crucial to collect datasets that represent the whole population and reflect its diversity as much as possible [5, 24, 25]. Yet clinical research often emphasizes the opposite: very homogeneous datasets and carefully selected participants. While this may help reduce variance and improve statistical testing, it degrades prediction performance and fairness. In other words, the machine-learning system may perform worse for segments of the population that are under-represented in the dataset, resulting in uneven quality of care if it is deployed in clinical settings. Therefore in *predictive* settings, where the goal is machine-learning models that generalize well, large and diverse datasets are desirable.

Simpler models are not less sensitive to dataset shift. Often, flexible models can be more robust to dataset shifts, and thus generalize better, than linear models [26], as seen in Figures 1 and 2. Indeed, an over-constrained (ill-specified) model may only fit well a restricted region of the feature space, and its performance can degrade if the distribution of inputs changes, even if the relation to the output stays the same (i.e. when covariate shift occurs, Section 7.1).

Dataset shift does not call for simpler models as it is not a small-sample issue. Collecting more data from the same sources will not correct systematic dataset bias.

4 Preferential sample selection: a common source of shift

In 2017, competitors in the million-dollar-prize data science bowl used machine learning to predict if individuals would be diagnosed with lung cancer within one year, based on a CT scan.

Assuming that the winning model achieves satisfying accuracy on left-out examples from this dataset, is it ready to be deployed in hospitals? Most likely not. Selection criteria may make this dataset not representative of the potential lung cancer patients general population. Selected participants verified many criteria, including being a smoker and not having recent medical problems such as pneumonia. How would the winning predictor perform on a more diverse population? For example, another disease could present features that the classifier could mistakenly take for signs of lung cancer. Beyond explicit selection criteria, many factors such as age, ethnicity, or socioeconomic status influence participation in biomedical studies [27, 28, 20, 29]. Not only can these shifts reduce overall predictive performance, they can also lead to discriminative clinical decisions for poorly represented populations [30, 31, 32, 33, 34].

The examples above are instances of preferential selection, which happens when members of the population of interest do not have equal probabilities of being included in the source dataset: the selection S is not independent of (X, Y). Preferential sample selection is ubiquitous and cannot always be prevented by careful study design [35]. It is therefore a major challenge to the identification of reliable and fair biomarkers. Beyond preferential sample selection, there are many other sources of dataset shifts, e.g. population changes over time, interventions such as the introduction of new diagnostic codes in Electronic Health Records [36], and the use of different acquisition devices.

4.1 The selection mechanism influences the type of dataset shift

The correction for a dataset shift depends on the nature of this shift, characterized by which and how distributions are modified [26]. Knowledge of the mechanism producing the dataset shift helps formulate hypotheses about distributions that remain unchanged in the target data [37, 38, Chap. 5].

Figure 3 illustrates this process with a simulated example of preferential sample selection. We consider the problem of predicting the volume Y of a tumor from features X extracted from contrast CT images. These features can be influenced not only by the tumor size, but also by the dosage of a contrast agent M. The first panel of Figure 3 shows a selection of data independent of the image and tumor volume: there is no dataset shift. In the second panel, selection depends on the CT image itself (for example images with a low signal-to-noise ratio are discarded). As selection is independent of the tumor volume Y given the image X, the distribution of images changes but the conditional distribution $P(Y \mid X)$ stays the same: we face a *covariate shift* (Section 7.1). The learned association remains valid. Moreover, reweighting examples to give more importance to those less likely to be selected can improve predictions for target data (Section 5), and it can be done with only *unlabelled* examples from the target data. In the third panel, individuals who received a low contrast agent dose are less likely to enter the training dataset. Selection is therefore not independent of tumor volume (the output) given the image values (the input features). Therefore we have sample selection bias: the relation $P(Y \mid X)$ is different in source and target data, which will affect the performance of the prediction.

As these examples illustrate, the causal structure of the data helps identify the type of dataset shift and what information is needed to correct it. When such information is available, it may be possible to leverage it in order to improve robustness to dataset shift [e.g. 40].

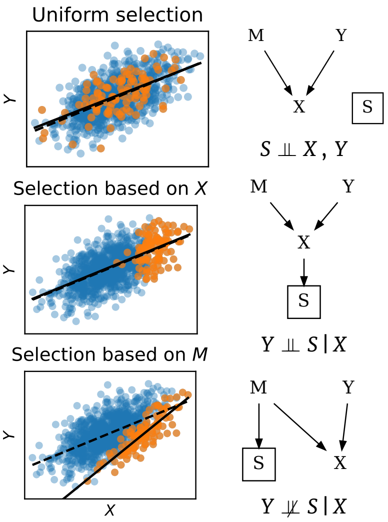

Figure 3. Sample selection bias: three examples. On the right are graphs giving conditional independence relations [39]. Y is the lesion volume to be predicted (i.e. the output). M are the imaging parameters, e.g. contrast agent dosage. X is the image, and depends both on Y and M (in this toy example X is computed as $X := Y + M + \epsilon$, where ϵ is additive noise. S indicates that data is selected to enter the source dataset (orange points) or not (blue points). The symbol $\perp\!\!\!\perp$ means independence between variables. Preferentially selecting samples results in a dataset shift (middle and bottom row). Depending on whether $Y \perp\!\!\!\perp S \mid X$, the conditional distribution of $Y \mid X$ – here lesion volume given the image – estimated on the selected data may be biased or not.

5 Importance weighting: a generic tool against dataset shift

Importance weighting is a simple approach to dataset shift that applies to many situations and can be easy to implement.

Dataset shift occurs when the joint distribution of the features and outputs is different in the source (data used to fit the machine-learning model) and in the target data. Informally, importance weighting consists in *reweighting* the available data to create a pseudo-sample that follows the same distribution as the target population.

To do so, examples are reweighted by their *importance weights* – the ratio of their likelihood in target data over source data. Examples that are rare in the source data but are likely in the target data are more relevant and therefore receive higher weights. A related approach is importance *sampling* – resampling the training data according to the importance weights. Many statistical learning algorithms – including Support Vector Machines, decision trees, random forests, neural networks – naturally support weighting the training examples. Therefore, the challenge relies mostly in the estimation of the appropriate sample weights and the learning algorithm itself does not need to be modified.

To successfully use importance weighting, no part of the target distribution should be completely unseen. For example, if sex (among other features) is used to predict heart failure and the dataset only includes men, importance weighting cannot transform this dataset and make its sex distribution similar to that of the general population (Figure 4). Conversely, the source distribution may be broader than the target distribution (as seen for example in Figure 1).

Importance weights can also be applied to validation examples, which may produce a more accurate estimation of generalization error on target data.

Importance weighting is a well-known approach and an important body of literature focuses on its application and the estimation of importance weights. It is illustrated on small datasets for the prediction of breast cancer in Dudík et al. [41] and heart

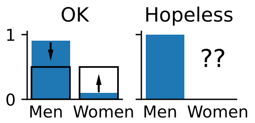

Figure 4. Dataset shifts that may or may not be compensated by reweighting – **Left:** distribution of sex can be balanced by downweighting men and upweighting women. **Right:** women are completely missing; the dataset shift cannot be fixed by importance weighting.

disease in Kouw and Loog [42]. However, it cannot always be applied: some knowledge of the target distribution is required, and the source distribution must cover its support. Moreover, importance weighting can increase the variance of the empirical risk estimate, and thus sometimes *degrades* performance – as seen in Figure 2. It is therefore a straightforward and popular approach to consider, but not a complete solution. It is particularly beneficial when using a simple learning model which cannot capture the full complexity of the data, such as the linear models in Figure 1. Indeed, simple models are often prefered in biomedical applications because they are easy to interpret and audit.

In Appendix A, we provide a more precise definition of the importance weights, as well as an overview of how they can be estimated and used.

6 Other approaches to dataset shift

Beyond importance weighting, many other solutions to dataset shift have been proposed. They are typically more difficult to implement, as they require adapting or desiging new learning algorithms. However, they may be more effective, or applicable when information about the target distribution is lacking. We summarize a few of these approaches here. A more systematic review can be found in Kouw and Loog [42]. Weiss et al. [43] and Pan and Yang [44] give systematic reviews of transfer learning (a wider family of learning problems which includes dataset shift).

The most obvious solution is to do nothing – ignoring the dataset shift. This approach should be included as a baseline when testing on a sample of target data – which is a prerequisite to clinical use of a biomarker [26, 11]. With flexible models, this is a strong baseline that can outperform importance weighting, as in the right panel of Figure 2.

Another approach is to learn representations–transformations of the signal— that are invariant to the shift [45]. Some deep-learning methods strive to extract features that are predictive of the target while having similar distributions in the source and target domains [e.g. 46], or while preventing an adversary to distinguish source and target data ["domain-adversarial" learning, e.g. 47]. When considering such methods, one must be aware of the fallacy shown in Figure 1: making the features invariant to the effect driving the dataset shift can remove valuable signal if this effect is not independent of the outcome of interest.

It may also be possible to explicitly model the mapping from source to target domains, e.g. by training a neural network to translate images from one modality or imaging device to another, or by relying on optimal transport [48].

Finally, synthetic data augmentation sometimes helps – relying on known invariances e.g. for images by applying affine transformations, resampling, *etc.*. or with learned generative models [e.g. 49].

6.1 Performance heterogeneity and fairness

It can be useful not to target a specific population, but rather find a predictor robust to certain dataset shifts. Distributionally robust optimization tackles this goal by defining an ambiguity, or uncertainty set – a set of distributions to which the target distribution might belong – then minimizing the worse risk across all distributions in this set [see 50, for a review]. The uncertainty set is often chosen centered on the empirical (source) distribution for some divergence between distributions. Popular choices for this divergence are the Wasserstein distance, f-divergences (e.g. the KL divergence) [51], and the Maximum Mean Discrepancy [52]. If information about the target distribution is available, it can be incorportated in the definition of the uncertainty set. An approach related to robust optimization is to strive not only to minimize the empirical loss $L(Y, f(X))$ but also its variance [53, 54].

It is also useful to assess model performance across values of demographic variables such as age, socioeconomic status or ethnicity. Indeed, a good overall prediction performance can be achieved despite a poor performance on a minority group. Ensuring that a predictor performs well for all subpopulations reduces sensitivity to potential shifts in demographics and is essential to ensure fairness [33]. For instance, there is a risk that machine-learning analysis of dermoscopic images under-diagnoses malignant moles on skin tones that are typically under-represented in the training set [55]. Fairness is especially relevant when the model output could be used to grant access to some treatment. As similar issues arise in many applications of machine learning, there is a growing literature on fairness [see e.g. 32, for an overview]. For instance, Duchi and Namkoong [51] show that distributionally robust optimization can help performance on under-represented subpopulations.

6.2 Multi-site datasets

Often datasets are collected across several sites or hospitals, or with different measurement devices. This heterogeneity provides an opportunity to train models that generalize to unseen sites or devices. Some studies attempt to remove site effects by regressing all features on the site indicator variable. For the same reasons that regressing out age is detrimental in Figure 1, this strategy often gives worse generalization across sites.

Data harmonization, such as compensating differences across measurement devices, is crucial, but remains very difficult and cannot correct these differences perfectly [56]. Removing too much inter-site variance can lead to loss of informative signal. Rather, it is important to model it well, accounting for the two sources of variance, across participants and across sites. A good model strives to yield good results on all sites. One solution is to adapt ideas from robust optimization: on data drawn from different distributions (e.g. from several sites), Krueger et al. [57] show the benefits of minimizing the empirical risk on the worse site or adding penalties on the variance of the loss across sites.

Measures of prediction performance should aggregate scores at the site level (not pooling all individuals), and check the variance across sites and the performance on the worse site. Cross-validation schemes should hold out entire sites [11, 58].

7 Special cases of dataset shift

Categorizing dataset shift helps finding the best approach to tackle it [26, 59]. We summarize two frequently-met scenarios that are easier to handle than the general case and can call for different adjustments: covariate shift (Section 7.1) and prior probability shift (Section 7.2).

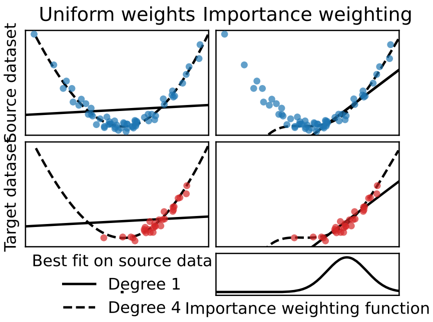

Figure 5. Covariate shift: $P(Y \mid X)$ stays the same but the feature space is sampled differently in the source and target datasets. A powerful learner may generalize well as $P(Y \mid X)$ is correctly captured [26]. Thus the polynomial fit of degree 4 performs well on the new dataset. However, an overconstrained learner such as the linear fit can benefit from reweighting training examples to give more importance to the most relevant region of the feature space.

7.1 Covariate shift

Covariate shift occurs when the marginal distribution of X changes between the source and target datasets (i.e. $p_t(x) \neq p_s(x)$), but $P(Y \mid X)$ stays the same. This happens for example in the second scenario in Figure 3, where sample selection based on X (but not Y) changes the distribution of the inputs. If the model is correctly specified, an estimator trained with uniform weights will lead to optimal predictions given sufficient training data [prediction consistency 60, Lemma 4]. However the usual (unweighted) estimator is not consistent for an over-constrained (misspecified) model. Indeed, a over-constrained model may be able to fit the data well only in some regions of the input feature space (Figure 1). In this case reweighting training examples (Section 5) to give more importance to those that are more representative of the target data is beneficial [26, 37]. Figure 5 illustrates covariate shift.

7.2 Prior probability shift

Another relatively simple case of dataset shift is *prior probability shift*. With prior probability shift (a.k.a. label shift or target shift), the distribution of Y changes but not $P(X \mid Y)$. This happens for example when disease prevalence changes in the target population but manifests itself in the same way. Even more frequently, prior probability shift arises when one rare class is over-represented in the training data so that the dataset is more balanced, as when extracting a biomarker from a case-control cohort, or when the dataset is resampled as a strategy to handle the *class imbalance* problem [61]. Prior probability shift can be corrected without extracting a new biomarker, simply by adjusting a model's predicted probabilities using Bayes' rule [as noted for example in 26, 37]. When the classes are well separated, the effect of this correction may be small, i.e. the uncorrected classifier may generalize well without correction. Figure 6 illustrates prior probability shift.

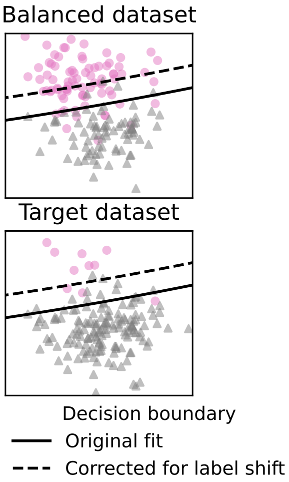

Figure 6. Prior probability shift: when $P(Y)$ changes but $P(X \mid Y)$ stays the same. This can happen for example when participants are selected based on Y – possibly to have a dataset with a balanced number of patients and healthy participants: $X \leftarrow Y \rightarrow \boxed{S}$. When the prior probability (marginal distribution of Y) in the target population is known, this is easily corrected by applying Bayes' rule. The output Y is typically low-dimensional and discrete (often it is a single binary value), so $P(Y)$ can often be estimated precisely from few examples.

8 Conclusion

Ideally, machine learning biomarkers would be designed and trained using datasets carefully collected to be representative of the targeted population – as in Liu et al. [62]. To be trusted, biomarkers ultimately need to be evaluated rigorously on one or several independent and representative samples. However, such data collection is expensive. It is therefore useful to exploit existing datasets in an opportunistic way as much as possible in the early stages of biomarker development. When doing so, correctly accounting for dataset shift can prevent wasting important resources on machine-learning predictors that have little chance of performing well outside of one particular dataset.

We gave an overview of importance weighting, a simple tool against dataset shift. Importance weighting needs a clear definition of the targeted population and access to a diverse training dataset. When this is not possible, distributionally robust optimization may be promising alternative, though it is a more recent approach and more difficult to implement. Despite much work and progress, dataset shift remains a difficult problem. Characterizing its impact and the effectiveness of existing solutions for biomarker discovery will be important for machine learning models to become more reliable in healthcare applications.

We conclude with the following recommendations:

- be aware of the dataset shift problem and the difficulty of out-of-dataset generalization. Do not treat cross-validation scores on one dataset as a guarantee that a model will perform well on clinical data.
- collect diverse, representative data.
- use powerful machine-learning models and large datasets.
- consider using importance weighting to correct biases in the data collection, especially if the learning model may be over-constrained (e.g. when using a linear model).
- look for associations between prediction performance and demographic variables in the validation set to detect potential generalization or fairness issues.
- *do not* remove confounding signal in a predictive setting.

These recommendations should help designing fair biomarkers and their efficient application on new cohorts.

Author contributions. Jérôme Dockès, Gaël Varoquaux and Jean-Baptiste Poline participated in conception, literature search, data interpretation, and editing the manuscript. Jérôme Dockès wrote the software and drafted the manuscript. Both Gaël Varoquaux and Jean-Baptiste Poline contributed equally to this work (as last authors).

Competing interests statement. The authors declare that there are no competing interests.

Software and data availability. The source files used to create this publication can be found in this repository: `https://github.com/neurodatascience/dataset_shift_biomarkers`. UKBiobank data can be obtained from `https://www.ukbiobank.ac.uk`.

References

1. Strimbu K, Tavel JA. What are biomarkers? Current Opinion in HIV and AIDS 2010;5(6):463.
2. Andreu-Perez J, Poon CC, Merrifield RD, Wong ST, Yang GZ. Big data for health. IEEE journal of biomedical and health informatics 2015;19(4):1193–1208.
3. Faust O, Hagiwara Y, Hong TJ, Lih OS, Acharya UR. Deep learning for healthcare applications based on physiological signals: A review. Computer methods and programs in biomedicine 2018;161:1–13.
4. Deo RC. Machine learning in medicine. Circulation 2015;132(20):1920–1930.
5. Kakarmath S, Esteva A, Arnaout R, Harvey H, Kumar S, Muse E, et al. Best practices for authors of healthcare-related artificial intelligence manuscripts. NPJ Digital Medicine 2020;.
6. Larrazabal AJ, Nieto N, Peterson V, Milone DH, Ferrante E. Gender imbalance in medical imaging datasets produces biased classifiers for computer-aided diagnosis. Proceedings of the National Academy of Sciences 2020;117:12592.
7. Rothman KJ. Epidemiology: an introduction. Oxford university press; 2012.
8. Tripepi G, Jager KJ, Dekker FW, Zoccali C. Selection bias and information bias in clinical research. Nephron Clinical Practice 2010;115(2):c94–c99.
9. Ransohoff DF, Feinstein AR. Problems of spectrum and bias in evaluating the efficacy of diagnostic tests. New England Journal of Medicine 1978;299(17):926–930.
10. Mulherin SA, Miller WC. Spectrum bias or spectrum effect? Subgroup variation in diagnostic test evaluation. Annals of internal medicine 2002;137(7):598–602.
11. Woo CW, Chang LJ, Lindquist MA, Wager TD. Building better biomarkers: brain models in translational neuroimaging. Nature neuroscience 2017;20(3):365.
12. Wynants L, Van Calster B, Bonten MM, Collins GS, Debray TP, De Vos M, et al. Prediction models for diagnosis and prognosis of covid-19 infection: systematic review and critical appraisal. bmj 2020;369.
13. Kasahara R, Kino S, Soyama S, Matsuura Y. Noninvasive glucose monitoring using mid-infrared absorption spectroscopy based on a few wavenumbers. Biomedical optics express 2018;9(1):289–302.
14. Friedman J, Hastie T, Tibshirani R. The elements of statistical learning. Springer series in statistics New York; 2001.
15. Poldrack RA, Huckins G, Varoquaux G. Establishment of best practices for evidence for prediction: a review. JAMA psychiatry 2020;77(5):534–540.
16. Arlot S, Celisse A, et al. A survey of cross-validation procedures for model selection. Statistics surveys 2010;4:40–79.
17. Beck AH, Sangoi AR, Leung S, Marinelli RJ, Nielsen TO, Van De Vijver MJ, et al. Systematic analysis of breast cancer morphology uncovers stromal features associated with survival. Science translational medicine 2011;3(108):108ra113–108ra113.
18. Jin D, Zhou B, Han Y, Ren J, Han T, Liu B, et al. Generalizable, Reproducible, and Neuroscientifically Interpretable Imaging Biomarkers for Alzheimer's Disease. Advanced Science 2020;p. 2000675.
19. Bleeker S, Moll H, Steyerberg E, Donders A, Derksen-Lubsen G, Grobbee D, et al. External validation is necessary in prediction research:: A clinical example. Journal of clinical epidemiology 2003;56(9):826–832.
20. Heiat A, Gross CP, Krumholz HM. Representation of the elderly, women, and minorities in heart failure clinical trials. Archives of internal medicine 2002;162(15).
21. Hernán M, Robins J. Causal inference: What if. Boca Raton: Chapman & Hill/CRC 2020;.
22. Pearl J. The seven tools of causal inference, with reflections on machine learning. Communications of the ACM 2019;62(3):54–60.
23. Sudlow C, Gallacher J, Allen N, Beral V, Burton P, Danesh J, et al. UK biobank: an open access resource for identifying the causes of a wide range of complex diseases of middle and old age. PLoS medicine 2015;12(3).
24. England JR, Cheng PM. Artificial intelligence for medical image analysis: a guide for authors and reviewers. American Journal of Roentgenology 2019;212(3):513–519.
25. O'neil C. Weapons of math destruction: How big data increases inequality and threatens democracy. Broadway Books; 2016.
26. Storkey A. When training and test sets are different: characterizing learning transfer. Dataset shift in machine learning 2009;p. 3–28.
27. Henrich J, Heine SJ, Norenzayan A. Most people are not WEIRD. Nature 2010;466(7302):29–29.
28. Murthy VH, Krumholz HM, Gross CP. Participation in cancer clinical trials: race-, sex-, and age-based disparities. Jama 2004;291(22):2720–2726.
29. Chastain DB, Osae SP, Henao-Martínez AF, Franco-Paredes C, Chastain JS, Young HN. Racial disproportionality in Covid clinical trials. New England Journal of Medicine 2020;383(9):e59.
30. Oakden-Rayner L, Dunnmon J, Carneiro G, Ré C. Hidden stratification causes clinically meaningful failures in machine learning for medical imaging. In: Proceedings of the ACM Conference on Health, Inference, and Learning; 2020. p. 151–159.
31. Gianfrancesco MA, Tamang S, Yazdany J, Schmajuk G. Potential biases in machine learning algorithms using electronic health record data. JAMA internal medicine 2018;178(11):1544–1547.
32. Barocas S, Hardt M, Narayanan A. Fairness and Machine Learning. fairmlbook.org; 2019. `http://www.fairmlbook.org`.
33. Abbasi-Sureshjani S, Raumanns R, Michels BEJ, Schouten G, Cheplygina V. Risk of Training Diagnostic Algorithms on Data with Demographic Bias. In: Interpretable and Annotation-Efficient Learning for Medical Image Computing Cham: Springer International Publishing; 2020. p. 183–192.
34. Cirillo D, Catuara-Solarz S, Morey C, Guney E, Subirats L, Mellino S, et al. Sex and gender differences and biases in artificial intelligence for biomedicine and healthcare. NPJ Digital Medicine 2020;3(1):1–11.
35. Bareinboim E, Pearl J. Controlling selection bias in causal inference. In: Artificial Intelligence and Statistics; 2012. p. 100–108.
36. Sáez C, Gutiérrez-Sacristán A, Kohane I, García-Gómez JM, Avillach P. EHRtemporalVariability: delineating temporal dataset shifts in electronic health records. medRxiv 2020;.
37. Schölkopf B, Janzing D, Peters J, Sgouritsa E, Zhang K, Mooij J. On causal and anticausal learning. In: 29th International Conference on Machine Learning (ICML 2012) International Machine Learning Society; 2012. p. 1255–1262.
38. Peters J, Janzing D, Schölkopf B. Elements of causal inference: foundations and learning algorithms. MIT press; 2017.
39. Pearl J, Glymour M, Jewell NP. Causal inference in statistics: A primer. John Wiley & Sons; 2016.

40. Subbaswamy A, Schulam P, Saria S. Preventing failures due to dataset shift: Learning predictive models that transport. In: The 22nd International Conference on Artificial Intelligence and Statistics; 2019. p. 3118–3127.
41. Dudík M, Phillips SJ, Schapire RE. Correcting sample selection bias in maximum entropy density estimation. In: Advances in neural information processing systems; 2006. p. 323–330.
42. Kouw WM, Loog M. A review of domain adaptation without target labels. IEEE transactions on pattern analysis and machine intelligence 2019;.
43. Weiss K, Khoshgoftaar TM, Wang D. A survey of transfer learning. Journal of Big data 2016;3(1):9.
44. Pan SJ, Yang Q. A survey on transfer learning. IEEE Transactions on knowledge and data engineering 2009;22(10):1345–1359.
45. Achille A, Soatto S. Emergence of invariance and disentanglement in deep representations. The Journal of Machine Learning Research 2018;19(1):1947–1980.
46. Long M, Cao Y, Wang J, Jordan M. Learning transferable features with deep adaptation networks. In: International conference on machine learning PMLR; 2015. p. 97–105.
47. Tzeng E, Hoffman J, Saenko K, Darrell T. Adversarial discriminative domain adaptation. In: Proceedings of the IEEE conference on computer vision and pattern recognition; 2017. p. 7167–7176.
48. Courty N, Flamary R, Tuia D, Rakotomamonjy A. Optimal transport for domain adaptation. IEEE transactions on pattern analysis and machine intelligence 2016;39(9):1853–1865.
49. Antoniou A, Storkey A, Edwards H. Data augmentation generative adversarial networks. arXiv preprint arXiv:171104340 2017;.
50. Rahimian H, Mehrotra S. Distributionally robust optimization: A review. arXiv preprint arXiv:190805659 2019;.
51. Duchi J, Namkoong H. Learning models with uniform performance via distributionally robust optimization. arXiv preprint arXiv:181008750 2018;.
52. Zhu JJ, Jitkrittum W, Diehl M, Schölkopf B. Kernel Distributionally Robust Optimization. arXiv preprint arXiv:200606981 2020;.
53. Maurer A, Pontil M. Empirical Bernstein Bounds and Sample Variance Penalization. stat 2009;1050:21.
54. Namkoong H, Duchi JC. Variance-based regularization with convex objectives. In: Advances in neural information processing systems; 2017. p. 2971–2980.
55. Adamson AS, Smith A. Machine learning and health care disparities in dermatology. JAMA dermatology 2018;154(11):1247–1248.
56. Glocker B, Robinson R, Castro DC, Dou Q, Konukoglu E. Machine learning with multi-site imaging data: An empirical study on the impact of scanner effects. arXiv preprint arXiv:191004597 2019;.
57. Krueger D, Caballero E, Jacobsen JH, Zhang A, Binas J, Priol RL, et al. Out-of-Distribution Generalization via Risk Extrapolation (REx). arXiv preprint arXiv:200300688 2020;.
58. Little MA, Varoquaux G, Saeb S, Lonini L, Jayaraman A, Mohr DC, et al. Using and understanding cross-validation strategies. Perspectives on Saeb et al. GigaScience 2017;6(5):gix020.
59. Moreno-Torres JG, Raeder T, Alaiz-RodríGuez R, Chawla NV, Herrera F. A unifying view on dataset shift in classification. Pattern recognition 2012;45(1):521–530.
60. Shimodaira H. Improving predictive inference under covariate shift by weighting the log-likelihood function. Journal of statistical planning and inference 2000;90(2):227–244.
61. He H, Garcia EA. Learning from imbalanced data. IEEE Transactions on knowledge and data engineering 2009;21(9):1263–1284.
62. Liu M, Oxnard G, Klein E, Swanton C, Seiden M, Liu MC, et al. Sensitive and specific multi-cancer detection and localization using methylation signatures in cell-free DNA. Annals of Oncology 2020;.
63. Zadrozny B, Langford J, Abe N. Cost-sensitive learning by cost-proportionate example weighting. In: Third IEEE international conference on data mining IEEE; 2003. p. 435–442.
64. Zadrozny B. Learning and evaluating classifiers under sample selection bias. In: Proceedings of the twenty-first international conference on Machine learning; 2004. p. 114.
65. Sugiyama M, Krauledat M, Müller KR. Covariate shift adaptation by importance weighted cross validation. Journal of Machine Learning Research 2007;8(May):985–1005.
66. Cortes C, Mohri M, Riley M, Rostamizadeh A. Sample selection bias correction theory. In: International conference on algorithmic learning theory Springer; 2008. p. 38–53.
67. Hernán MA, Hernández-Díaz S, Robins JM. A structural approach to selection bias. Epidemiology 2004;p. 615–625.
68. Austin PC. An introduction to propensity score methods for reducing the effects of confounding in observational studies. Multivariate behavioral research 2011;46(3):399–424.
69. Sugiyama M, Kawanabe M. Machine learning in non-stationary environments: Introduction to covariate shift adaptation. MIT press; 2012.
70. Sun B, Feng J, Saenko K. Return of frustratingly easy domain adaptation. In: Thirtieth AAAI Conference on Artificial Intelligence; 2016. .
71. Huang J, Gretton A, Borgwardt K, Schölkopf B, Smola AJ. Correcting sample selection bias by unlabeled data. In: Advances in neural information processing systems; 2007. p. 601–608.
72. Zhang K, Schölkopf B, Muandet K, Wang Z. Domain adaptation under target and conditional shift. In: International Conference on Machine Learning; 2013. p. 819–827.
73. Sugiyama M, Nakajima S, Kashima H, Buenau PV, Kawanabe M. Direct importance estimation with model selection and its application to covariate shift adaptation. In: Advances in neural information processing systems; 2008. p. 1433–1440.
74. Kanamori T, Hido S, Sugiyama M. A least-squares approach to direct importance estimation. The Journal of Machine Learning Research 2009;10:1391–1445.
75. Niculescu-Mizil A, Caruana R. Predicting good probabilities with supervised learning. In: Proceedings of the 22nd international conference on Machine learning; 2005. p. 625–632.

A Definition and estimation of importance weights

We will implicitly assume that all the random variables we consider admit densities and denote p_s and p_t the density of the joint distribution of (X, Y) applied to the source and target populations respectively. If the support of p_t is included in that of p_s (meaning that $p_s > 0$ wherever $p_t > 0$), we have:

$$\mathbb{E}_{\mathrm{source}}[\, L(Y, f(X))\,] = \mathbb{E}_{\mathrm{target}}\left[\frac{p_t(X, Y)}{p_s(X, Y)}\, L(Y, f(X))\right] , \quad (3)$$

where L is the cost function and f is a prediction function, $\mathbb{E}_{\mathrm{source}}$ (resp. $\mathbb{E}_{\mathrm{target}}$) the expectation on the source (resp. target) data. The risk (on target data) can therefore be computed as an expectation on the source distribution where the loss function is reweighted by the *importance weights*:

$$w(x, y) = \frac{p_t(x, y)}{p_s(x, y)} . \quad (4)$$

If $\hat{w}$ are empirical estimates of the importance weights w, it is possible to compute the reweighted empirical risk:

$$\hat{R}_{\hat{w}}(f) = \sum_{i=1}^{n} \hat{w}(x_i, y_i)\, L(y_i, f(x_i)) \,. \tag{5}$$

Rather than being weighted, examples can also be resampled with importance or rejection sampling [63, 64]. Importances can also be taken into account for model selection – for example in Sugiyama et al. [65] examples of the test set are also reweighted when computing cross-validation scores. Cortes et al. [66] study how errors in the estimation of the weights affect the prediction performance.

A.1 Preferential Sample selection and Inverse Probability weighting

In the case of preferential sample selection (Section 4), the condition that requires for the support of p_t to be included in the support of p_s translates to a requirement that all individuals have a non-zero probability of being selected: $P(S = 1 \mid x, y) > 0$ for all (x, y) in the support of p_t. When this is verified, by applying Bayes' rule the definition of importance weights in Equation (4) can be reformulated [see 66, Sec. 2.3]:

$$w(x, y) = \frac{P(S = 1)}{P(S = 1 \mid X = x, Y = y)} \tag{6}$$

These weights are sometimes called Inverse Probability weights [67] or Inverse Propensity scores [68]. Training examples that had a low probability of being selected receive higher weights, because they have to account for similar individuals who were not selected.

A.2 Computing importance weights

In practice $p_t(x, y)$, which is the joint density of (X, Y) in the target data, is not known. However, it is not needed for the estimation of p_t/p_s. More efficient estimation hinges on two observations: estimation of both densities separately is not necessary to estimate their ratio, and variables that have the same distribution in source and target data can be factored out.

Here we describe methods that estimate the true importance weights p_t/p_s, but we point out that reweighting the training examples reduces the bias of the empirical risk but increases the variance of the estimated model parameters. Even when the importances are perfectly known, it can therefore be beneficial to regularize the weights [60].

Computing importance weights does not require distributions densities estimation

Importance weights can be computed by modelling separately p_s and p_t and then computing their ratio [69, Sec. 4.1]. However, distribution density estimation is notoriously difficult; non-parametric methods suffer from the curse of dimensionality and parametric methods depend heavily on the correct specification of a parametric form.

But estimating both densities is more information than is needed to compute the sample weights. Instead, one can directly optimize importance weights in order to make the reweighted sample similar to the target distribution, by matching moments [70] or mean embeddings [71, 72], minimizing the KL-divergence [73], solving a least-squares estimation problem [74] or with optimal transport [48].

Alternatively, a discriminative model can be trained to distinguish source and target examples. In the specific case of preferential sample selection, this means estimating directly the probability of selection $P(S = 1)$ (cf Equation (6)). In general, the shift is not always due to selection: the source data is not necessarily obtained by subsampling the target population. In this case we denote $T = 1$ if an individual comes from the target data and $T = 0$ if it comes from the source data. Then, a classifier can be trained to predict from which dataset (source or target) a sample is drawn, and the importance weights obtained from the predicted probabilities [69, Sec. 4.3]:

$$w(x, y) = \frac{P(T = 1 \mid X = x, Y = y)\, P(T = 0)}{P(T = 0 \mid X = x, Y = y)\, P(T = 1)} \,, \tag{7}$$

The classifier must be calibrated (i.e. produce accurate probability estimates, not only a correct decision), see Niculescu-Mizil and Caruana [75]. Note that constant factors such as $P(T = 0)/P(T = 1)$ usually do not matter and are easy to estimate if needed. This discriminative approach is effective because the distribution of $(T \mid X = x, Y = y)$ is much easier to estimate than the distribution of $(X, Y \mid T = t)$: T is a single binary variable whereas (X, Y) is high-dimensional and often continuous.

The classifier does not need to distinguish source and target examples with high accuracy. In the ideal situation of no dataset shift, the classifier will perform at chance level. On the contrary, a high accuracy means that there is little overlap between the source and target distributions and the model will probably not generalize well.

What distributions differ in source and target data?

When computing importance weights, it is possible to exploit prior knowledge that some distributions are left unchanged in the target data. For example,

$$\frac{p_t(x, y)}{p_s(x, y)} = \frac{p_t(y \mid x)\, p_t(x)}{p_s(y \mid x)\, p_s(x)} \,. \tag{8}$$

Imagine that the marginal distribution of input X differs in source and target data, but the conditional distribution of the output Y given the input stays the same: $p_t(x) \neq p_s(x)$ but $p_t(y \mid x) = p_s(y \mid x)$ (a setting known as *covariate shift*). Then, the importance weights simplify to

$$w(x, y) = \frac{p_t(x)}{p_s(x)} \,. \tag{9}$$

In this case, importance weights can be estimated using only unlabelled examples (individuals for whom Y is unkown) from the target distribution.

Often, the variables that influence selection (e.g. demographic variables such as age) are lower-dimensional than the full features (e.g. high-dimensional images), and dataset shift can be corrected with limited information on the target distribution, with importance weights or otherwise. Moreover, even if additional information Z that predicts selection but is independent of (X, Y) is available, it should *not* be used to compute the importance weights. Indeed, this would only increase the weights' variance without reducing the bias due to the dataset shift [21, Sec. 15.5].

B Tobacco smoking prediction in the UK-Biobank

We consider predicting the smoking status of participants in the UKBiobank study to illustrate the effect of dataset shift on prediction performance.

6,000 participants are used in a preliminary step to identify the 29 most relevant predictive features (listed in appendix B.1),

by cross-validating a gradient boosting model and computing permutation feature importances. We then draw two samples of 100K individuals from the rest of the dataset, that have different age distributions. In the young sample, 90% of individuals come from the youngest 20% of the dataset, and the remaining 10% are sampled from the oldest 20% of the dataset. In the old sample, these proportions are reversed. We then perform 10-fold cross validation. For each fold, both the training and testing set can be drawn from either the young or the old population, resulting in four tasks on which several machine-learning estimators are evaluated. We use this experiment to compare 2 machine-learning models: a simple one – regularized linear Support Vector Classifier, and a flexible one – Gradient Boosting. For each classifier, 3 strategies are considered to handle the dataset shift: (i) baseline – the generic algorithm without modifications, (ii) Importance Weighting (Section 5), and (iii) the (unfortunately popular) non-solution: "regressing out the confounder" – regressing the predictive features on the age and using the residuals as inputs to the classifier.

The results are similar to those seen with simulated data in Figure 1. For a given learner and test population, training on a different population degrades the prediction score. For example, if the learner is to be tested on the young population, it performs best when trained on the young population. Gradient Boosting vastly outperforms the linear model in all configurations. Regressing out the age always degrades the prediction; it is always worse than the unmodified baseline, whether a dataset shift is present or not. Finally, Importance Weighting (Section 5) improves the predictions of the over-constrained (misspecified) linear model in the presence of dataset shift, but degrades the prediction of the powerful learner used in this experiment. This is due to the fact that the Gradient Boosting already captures the correct separation for both young and old individuals, and therefore Importance Weighting does not bring any benefit but only reduces the effective training sample size by increasing the variance of the empirical risk.

B.1 Features used for tobacco smoking status prediction

The 30 most important features were identified in a preliminary experiment with 6,000 participants (that were not used in the subsequent analysis). One of these features, "Date F17 first reported (mental and behavioural disorders due to use of tobacco)", was deemed trivial – too informative, as it directly implies that the participant does smoke tobacco, and removed. The remaining 29 features were used for the experiment described in Section 3.

- Forced expiratory volume in 1-second (FEV1), predicted percentage
- Lifetime number of sexual partners
- Age first had sexual intercourse
- Age when last took cannabis
- Ever taken cannabis
- Forced expiratory volume in 1-second (FEV1), predicted
- Acceptability of each blow result
- Mouth/teeth dental problems
- Coffee intake
- FEV1/ FVC ratio Z-score
- Alcohol intake frequency.
- Date J44 first reported (other chronic obstructive pulmonary disease)
- Former alcohol drinker
- Average weekly spirits intake
- Year of birth
- Acceptability of each blow result
- Date of chronic obstructive pulmonary disease report
- Leisure/social activities
- Morning/evening person (chronotype)
- Mean sphered cell volume
- Lymphocyte count
- Townsend deprivation index at recruitment
- Age hay fever, rhinitis or eczema diagnosed
- Age started oral contraceptive pill
- White blood cell (leukocyte) count
- Age completed full time education
- Age at recruitment
- Workplace had a lot of cigarette smoke from other people smoking
- Wheeze or whistling in the chest in last year

C Glossary

Here we provide a summary of some terms and notations used in the paper.

Target population the population on which the biomarker (machine-learning model) will be applied.

Source population the population from which the sample used to train the machine-learning model is drawn.

Selection in the case that source data are drawn (with non-uniform probabilities) from the target population, we denote by $S = 1$ the fact that an individual is selected to enter the source data (e.g. to participate in a medical study).

Provenance of an individual when samples from both the source and the target populations (e.g. Appendix A.2) are available, we also denote $T = 1$ if an individual comes from the target population and $T = 0$ if they come from the source population.

Confounding in *causal inference*, when estimating the effect of a treatment on an outcome, confounding occurs if a third variable (e.g. age, a comorbidity, the seriousness of a condition) influences both the treatment and the outcome, possibly producing a spurious statistical association between the two. This notion is not directly relevant to dataset shift, and we mention it only to insist that it is a different problem. See Hernán and Robins [21], Chap. 7, for a more precise definition.

Domain adaptation the task of designing machine-learning methods that are resilient to dataset shift – essentially a synonym for dataset shift, i.e. another useful search term for readers looking for further information on this problem.
