# Supplementary material for: Preventing dataset shift from breaking machine-learning biomarkers
Source: Gigascience. 2021 Sep 28;10(9):giab055. doi: 10.1093/gigascience/giab055 (PMC8478611; doi:10.1093/gigascience/giab055)
Supplement: giab055_Response_to_Reviewer_Comments_Original_Submission [file giab055_response_to_reviewer_comments_original_submission.pdf]

Author's Response To Reviewer Comments

Close

To make it easier to read, we have prepared our response to reviewer and editor comments as a pdf file, attached to the new submission -- reply.pdf

Close
