# Supplementary material for: Preventing dataset shift from breaking machine-learning biomarkers
Source: Gigascience. 2021 Sep 28;10(9):giab055. doi: 10.1093/gigascience/giab055 (PMC8478611; doi:10.1093/gigascience/giab055)
Supplement: giab055_Reviewer_1_Report_Original_Submission — Guray Erus -- 5/10/2021 Reviewed [file giab055_reviewer_1_report_original_submission.pdf]

Reviewer Report

Title: Preventing dataset shift from breaking machine-learning biomarkers

Version: Original Submission **Date:** 5/10/2021

Reviewer name: Guray Erus

Reviewer Comments to Author:

This paper addresses a very important and often undermined challenge in deriving new biomarkers for disease using machine learning techniques. Very often, new methods are limited to validation experiments with cross-validation or using training and testing datasets with similar characteristics. Also, datasets used in validations are often affected by selection bias. Thus, these experiments may not provide a realistic evaluation of application to new individuals, e.g. in a clinical setting. The paper describes possible biases in data used for training and testing, describes the effects of "dataset shift" on the accuracy of final biomarkers, and presents techniques to deal with dataset shift.
The paper is well written in general. I enjoyed reading it as a tutorial that briefly presents the basic concepts and then incrementally introduces the main problem. The illustration of the dataset shift problem using toy examples and visualizations is very useful. After a very clear introduction and problem description, the paper presents a generic tool to address this problem. The proposed solution, importance weighting, is not novel. However, presenting it in this context was informative. Section 6.1 (covariate shift) nicely links to importance weighting technique. However, I found that section 6.2 was disconnected, so maybe it would require a more clear description and a careful discussion.
A few major comments:
- The paper reads well as a concept paper; however, it does not include any examples with real data. Toy examples for illustrations are very informative. However, examples on real datasets with quantitative evaluations would be necessary to show the effects of dataset shift in real problems, and to show how the
proposed approach actually works. I think that this is the major missing part in the paper. Addition of results using real datasets would significantly increase the value of the paper, particularly if they can be selected in a way that will illustrate the problems mentioned in the paper.
- A major challenge in deriving imaging biomarkers is to handle heterogeneity of imaging data and clinical labels due to various factors, such as scan parameters/protocols and variability of measuring protocols used. The paper did not discuss how to handle data heterogeneity, which is another major source for dataset shift. Only in section 3 there is a suggestion to use heterogeneous sets for training (which I agree), but it's not clear how to derive robust biomarkers in the presence of heterogeneous datasets (e.g. there is no mention of data harmonization). I think this is a limitation for the paper.
One minor comment that may help to improve the paper:
- In section 3, after reading the first sentence, I had the impression that the listed items in italic were the "misconceptions", so they are showing what is wrong/incorrect. I had to go back and read them again after I noticed that it was the opposite. I would suggest the authors to edit this part in a way that will remove the ambiguity.

Methods

Are the methods appropriate to the aims of the study, are they well described, and are necessary controls included? Choose an item.

Conclusions

Are the conclusions adequately supported by the data shown? Choose an item.

Reporting Standards

Does the manuscript adhere to the journal's guidelines on [minimum standards of reporting?] Choose an item.

Choose an item.

Statistics

Are you able to assess all statistics in the manuscript, including the appropriateness of statistical tests used? Choose an item.

Quality of Written English

Please indicate the quality of language in the manuscript: Choose an item.

Declaration of Competing Interests

Please complete a declaration of competing interests, considering the following questions:

- Have you in the past five years received reimbursements, fees, funding, or salary from an organisation that may in any way gain or lose financially from the publication of this manuscript, either now or in the future?
- Do you hold any stocks or shares in an organisation that may in any way gain or lose financially from the publication of this manuscript, either now or in the future?
- Do you hold or are you currently applying for any patents relating to the content of the manuscript?
- Have you received reimbursements, fees, funding, or salary from an organization that holds or has applied for patents relating to the content of the manuscript?
- Do you have any other financial competing interests?
- Do you have any non-financial competing interests in relation to this paper?

If you can answer no to all of the above, write 'I declare that I have no competing interests' below. If your reply is yes to any, please give details below.

I declare that I have no competing interests

I agree to the open peer review policy of the journal. I understand that my name will be included on my report to the authors and, if the manuscript is accepted for publication, my named report including any attachments I upload will be posted on the website along with the authors' responses. I agree for my report to be made available under an Open Access Creative Commons CC-BY license (http://creativecommons.org/licenses/by/4.0/). I understand that any comments which I do not wish to be included in my named report can be included as confidential comments to the editors, which will not be published.

Choose an item.

To further support our reviewers, we have joined with Publons, where you can gain additional credit to further highlight your hard work (see: https://publons.com/journal/530/gigascience). On publication of this paper, your review will be automatically added to Publons, you can then choose whether or not to claim your Publons credit. I understand this statement.

Yes Choose an item.
