# Supplementary material for: Preventing dataset shift from breaking machine-learning biomarkers
Source: Gigascience. 2021 Sep 28;10(9):giab055. doi: 10.1093/gigascience/giab055 (PMC8478611; doi:10.1093/gigascience/giab055)
Supplement: giab055_Reviewer_2_Report_Original_Submission — Spencer Thomas -- 5/13/2021 Reviewed [file giab055_reviewer_2_report_original_submission.pdf]

Reviewer Report

Title: Preventing dataset shift from breaking machine-learning biomarkers

Version: Original Submission **Date:** 5/13/2021

Reviewer name: Spencer Thomas

Reviewer Comments to Author:

This article covers a vitally important topic in machine learning generally and specifically its application to healthcare and life science. The mismatching of attributes and properties in training and testing data is a significant issue. The authors raise these important issues and present some ideas and methods for how to address what they call 'dataset drift'. Figure 3 and the corresponding text are of interest and this should be emphasized more than it is currently.

There are several areas where the paper can be improved and given the importance of the topic and target audience, I would strongly recommend the authors consider these changes.

The authors present some examples of dataset drift and possible issues that arise, some from the literature and some from toy examples. I think this would have much stronger impact if a real dataset were used in the paper to demonstrate this.

The authors refer to 'probability shift' to refer to the difference in populations sizes in the training and testing data, commonly referred to as class-imbalance. This is quite brief in the paper and constitutes one of the biggest issues in machine learning in life sciences and more emphasis should be devoted to this. Explicit refer to class-imbalance (and some references) is required here as there is a large area of research devoted to this. Moreover, the authors must be clear what are the disadvantages of training models on balanced data when the population is imbalanced, and how using training data that reflects the population (e.g. with impedance) and then using the proposed methods provide an advantage. Figure 6 could be extended to show this for example. The machine learning community often use balanced training data to avoid 'short cuts' to high accuracies and identify the features need to predict a label given the input. If you have an appropriate model that identifies robust features in training, then the low frequency of a class in the testing / real world data (e.g. rare disease) should not degreed performance. This may also apply to other characteristics / properties in the training data.

Similarity, the authors have not discussed the notion of transforming the data distributions prior to application of a model, e.g. optimal transport. What are the benefits of the proposed methods over these?

The authors state on Line 166 Training examples should not be selected to be homogeneous. This maybe conflating issues from each of the healthcare and ML domains and may not be a general recommendation for all problems, the authors should expand this discussion to justify this recommendation.

The authors often refer to the f(x) as the biomarker, this is not correct. F(x) is the model and the biomarkers are the inputs x. The model finds a combination of these to differentiate the classes through f(x). This leads to data (i.e. biomarker) vs model considerations which are not discussed. That is, models will be sensitive to 'dataset shifts', but if biomarkers are robust then they should be invariant to such

shifts (at least in theory) with a 'good' model. Additionally, you do not 'build biomarkers', the model identifies them in the data.
Section 5 why has the precise definition and overview in the appendix? This section required more detail as it is currently conceptual only. Further details can be in the appendix, but more are required here.
Some minor comments to the authors
Section 2.1 you use lower case x and y but have not defined them (as individual instances in X and Y). one small sentence will suffice, you do this in line 89 but this should be earlier. You also use X and Y for the seen and unseen data on line 83.
The first entice in section 2.2 line 111 does not read well and the citation doesn't relate to a statement clearly. Training performance only is not just an 'optimistic' estimate it is potentially meaningless due to what you are calling data shit and the fact that some ML methods (eg neural nets) can fit any arbitrary data and hence overfit.
Fig 1 caption. Age is indicated by shade not colour. Healthy and disease are indicated by colour. Also I assume blue corresponds 'unhealthy' patients as this is not stated and needs to be. It seems that the RBF-SVM could be improved for the source data (for younger patients)
Fig 2 the caption needs more information. what is the shade of the arrows representing? A gradient between younger and older? What to the arrows (and their width represent?) what is the joining arrow indicating?
The text should refer to figures in order, currently it refers to figures 1,5,3 … and figure 2 is first referred in figure 1's caption.
line 288-289 are not relevant to the rest of the paragraph. You have not mentioned anything to do with transfer learning.
Figure 4 is unnecessary as this is described in the text clearly.

Methods

Are the methods appropriate to the aims of the study, are they well described, and are necessary controls included? Choose an item.

Conclusions

Are the conclusions adequately supported by the data shown? Choose an item.

Reporting Standards

Does the manuscript adhere to the journal's guidelines on [minimum standards of reporting?](#) Choose an item.

Choose an item.

Statistics

Are you able to assess all statistics in the manuscript, including the appropriateness of statistical tests used? Choose an item.

Quality of Written English

Please indicate the quality of language in the manuscript: Choose an item.

Declaration of Competing Interests

Please complete a declaration of competing interests, considering the following questions:

- Have you in the past five years received reimbursements, fees, funding, or salary from an organisation that may in any way gain or lose financially from the publication of this manuscript, either now or in the future?
- Do you hold any stocks or shares in an organisation that may in any way gain or lose financially from the publication of this manuscript, either now or in the future?
- Do you hold or are you currently applying for any patents relating to the content of the manuscript?
- Have you received reimbursements, fees, funding, or salary from an organization that holds or has applied for patents relating to the content of the manuscript?
- Do you have any other financial competing interests?
- Do you have any non-financial competing interests in relation to this paper?

If you can answer no to all of the above, write 'I declare that I have no competing interests' below. If your reply is yes to any, please give details below.

I declare that I have no competing interests

I agree to the open peer review policy of the journal. I understand that my name will be included on my report to the authors and, if the manuscript is accepted for publication, my named report including any attachments I upload will be posted on the website along with the authors' responses. I agree for my report to be made available under an Open Access Creative Commons CC-BY license (http://creativecommons.org/licenses/by/4.0/). I understand that any comments which I do not wish to be included in my named report can be included as confidential comments to the editors, which will not be published.

Choose an item.

To further support our reviewers, we have joined with Publons, where you can gain additional credit to further highlight your hard work (see: https://publons.com/journal/530/gigascience). On publication of this paper, your review will be automatically added to Publons, you can then choose whether or not to claim your Publons credit. I understand this statement.

Yes Choose an item.
