# Supplementary material for: Preventing dataset shift from breaking machine-learning biomarkers
Source: Gigascience. 2021 Sep 28;10(9):giab055. doi: 10.1093/gigascience/giab055 (PMC8478611; doi:10.1093/gigascience/giab055)
Supplement: giab055_Reviewer_3_Report_Original_Submission — Enzo Ferrante -- 5/15/2021 Reviewed [file giab055_reviewer_3_report_original_submission.pdf]

**Reviewer Report**

**Title: Preventing dataset shift from breaking machine-learning biomarkers**

**Version: Original Submission Date:** 5/15/2021

**Reviewer name: Enzo Ferrante**

**Reviewer Comments to Author:**

This manuscript presents a didactic review which discusses the implications of dataset shift when designing and evaluating machine learning-based biomarkers. The review covers some basic machine learning concepts (empirical risk minimization, evaluation practices, etc), points out common misconceptions, characterizes different types of dataset shift, and discusses importance weighting as a potential solution to this problem.

The paper is nicely written, didactic and clear. I feel it is also timely since currently many researchers who are not coming from the fields of statistics or machine learning are using such methods to analyze their own datasets and derive biomarkers for various pathologies. Overall, the manuscript is interesting but I think it would benefit from including a few more concrete examples, linking the theoretical concepts to applications in the context of biomarkers (see some of my comments below).

I have some recommendations that may help to improve the quality of the paper:

- One common source of dataset shift in the context of image-derived biomarkers is related to the equipment brand or configuration parameters used to capture such images (e.g. the MR or CT machine used to perform the studies). I would like the authors to discuss this fact and link it to the concepts introduced in the review.
- To me, the target audience for this didactic review is mostly healthcare and biomedical researchers, who are using machine learning and data analysis tools to produce novel biomarkers. In that sense, I would not take for granted the fact that the audience is familiar with basic concepts like 'confounding' for example, which are used but not defined. Another important term which is used but not discussed is 'fairness' (line 176). Since this is an educational review, I recommend the authors to devote a few lines to
introduce such concepts and discuss them in the context of predictive models.
- The thick arrows in Figure 2 are a bit confusing. What does the direction of the thick arrows indicate?. Also, g and h are not specified. The Figure just indicates "for some g,h". What do g and h represent? What is their actual form?
- The authors focus on importance weighting as a tool to deal with dataset shift, and dedicate a complete appendix to its definition. However, as discussed in section 7, importance weighting needs a clear definition of the targeted population and access to a diverse dataset, which may not be the case in real scenarios. Since this review is related to biomarkers, I think it would be important to discuss a few more concrete and real scenarios (beyond the examples shown in the figures) where importance weighting could be used to mitigate dataset shift in the context of machine-learning based biomarkers.
- As previously discussed, importance weighting is somehow limited in situations where we do not have a clear definition of the targeted population. In fact, the authors discuss in the Conclusions section

alternative approaches that may be used when this is not possible (e.g. distributionally robust optimization). Why not devoting a complete section to discuss alternative approaches useful in the absence of information about the target population? I am not sure if the Conclusions section is the right place to introduce them.

- As far as I understand, all the examples provided in the manuscript are coming from synthetic data. I think for a didactic review like this one, and specially for a journal like Gigascience, it would be nice to provide a case study using real data, which reflects a dataset shift between source and target, that can be corrected using the discussed importance weighting strategy. It doesn't have to be a huge dataset, just a simple case with concrete features X and labels Y. Maybe using some public database of tabulated samples which illustrates a real scenario?

General Note: I think it is important to acknowledge the fact that I am coming from the computer vision and medical image computing community, not from statistics or causal analysis, and thus my comments in that regard may be limited.

Minor comment:

- Line 385: "Importance weighting needs a clear definition the targeted population". It should be "Importance weighting needs a clear definition of the targeted population".

**Methods**

Are the methods appropriate to the aims of the study, are they well described, and are necessary controls included? Choose an item.

**Conclusions**

Are the conclusions adequately supported by the data shown? Choose an item.

**Reporting Standards**

Does the manuscript adhere to the journal's guidelines on minimum standards of reporting? Choose an item.

Choose an item.

**Statistics**

Are you able to assess all statistics in the manuscript, including the appropriateness of statistical tests used? Choose an item.

**Quality of Written English**

Please indicate the quality of language in the manuscript: Choose an item.

**Declaration of Competing Interests**

Please complete a declaration of competing interests, considering the following questions:

- Have you in the past five years received reimbursements, fees, funding, or salary from an organisation that may in any way gain or lose financially from the publication of this manuscript, either now or in the future?
- Do you hold any stocks or shares in an organisation that may in any way gain or lose financially from the publication of this manuscript, either now or in the future?
- Do you hold or are you currently applying for any patents relating to the content of the manuscript?
- Have you received reimbursements, fees, funding, or salary from an organization that holds or has applied for patents relating to the content of the manuscript?
- Do you have any other financial competing interests?
- Do you have any non-financial competing interests in relation to this paper?

If you can answer no to all of the above, write 'I declare that I have no competing interests' below. If your reply is yes to any, please give details below.

I declare that I have no competing interests

I agree to the open peer review policy of the journal. I understand that my name will be included on my report to the authors and, if the manuscript is accepted for publication, my named report including any attachments I upload will be posted on the website along with the authors' responses. I agree for my report to be made available under an Open Access Creative Commons CC-BY license (http://creativecommons.org/licenses/by/4.0/). I understand that any comments which I do not wish to be included in my named report can be included as confidential comments to the editors, which will not be published.

Choose an item.

To further support our reviewers, we have joined with Publons, where you can gain additional credit to further highlight your hard work (see: https://publons.com/journal/530/gigascience). On publication of this paper, your review will be automatically added to Publons, you can then choose whether or not to claim your Publons credit. I understand this statement.

Yes Choose an item.
